# Rapid Detection of Bacterial Pathogens and Antimicrobial Resistance Genes in Clinical Urine Samples With Urinary Tract Infection by Metagenomic Nanopore Sequencing

**DOI:** 10.3389/fmicb.2022.858777

**Published:** 2022-05-17

**Authors:** Lei Zhang, Wenhua Huang, Shengwei Zhang, Qian Li, Ye Wang, Ting Chen, Hua Jiang, Decong Kong, Qingyu Lv, Yuling Zheng, Yuhao Ren, Peng Liu, Yongqiang Jiang, Ying Chen

**Affiliations:** ^1^College of Life Science, Yantai University, Yantai, China; ^2^State Key Laboratory of Pathogen and Biosecurity, Beijing Institute of Microbiology and Epidemiology, Academy of Military Medical Sciences, Beijing, China; ^3^Department of Clinical Laboratory, Dongfang Hospital, Beijing University of Chinese Medicine, Beijing, China

**Keywords:** urinary tract infections (UTIs), metagenomics, Illumina sequencing, nanopore sequencing, diagnosis, antimicrobial resistance

## Abstract

Urinary tract infections (UTIs) are among the most common acquired bacterial infections in humans. The current gold standard method for identification of uropathogens in clinical laboratories is cultivation. However, culture-based assays have substantial drawbacks, including long turnaround time and limited culturability of many potential pathogens. Nanopore sequencing technology can overcome these limitations and detect pathogens while also providing reliable predictions of drug susceptibility in clinical samples. Here, we optimized a metagenomic nanopore sequencing (mNPS) test for pathogen detection and identification in urine samples of 76 patients with acute uncomplicated UTIs. We first used twenty of these samples to show that library preparation by the PCR Barcoding Kit (PBK) led to the highest agreement of positive results with gold standard clinical culture tests, and enabled antibiotic resistance detection in downstream analyses. We then compared the detection results of mNPS with those of culture-based diagnostics and found that mNPS sensitivity and specificity of detection were 86.7% [95% confidence interval (CI), 73.5–94.1%] and 96.8% (95% CI, 82.4–99.9%), respectively, indicating that the mNPS method is a valid approach for rapid and specific detection of UTI pathogens. The mNPS results also performed well at predicting antibiotic susceptibility phenotypes. These results demonstrate that our workflow can accurately diagnose UTI-causative pathogens and enable successful prediction of drug-resistant phenotypes within 6 h of sample receipt. Rapid mNPS testing is thus a promising clinical diagnostic tool for infectious diseases, based on clinical urine samples from UTI patients, and shows considerable potential for application in other clinical infections.

## Introduction

Urinary tract infections (UTIs) are one of the most prevalent diseases worldwide, affecting more than 150 million people annually (Terlizzi et al., 2017; Ujmajuridze et al., 2018). In severe cases, infection can spread to the kidneys, invade the bloodstream, and cause uremia and bacteremia (Rosen et al., 2007; Schmidt et al., 2017; Neugent et al., 2020; Kaushik et al., 2021). Early and appropriate antibiotic therapy is essential for control of UTIs. However, therapeutic management can be challenging due to the emergence of multidrug resistant (MDR) bacteria and the increasing rates of their resistance, especially that produced by extended-spectrum β-lactamases (ESBL) or carbapenemases in Gram-negative bacteria (Barraud et al., 2014, 2019; Lemon et al., 2017; Finton et al., 2020). Antimicrobial resistance has thus become a predominant public health challenge in recent decades (Pedroso et al., 2017; Hendriksen et al., 2019; McAdams et al., 2019; Aytan-Aktug et al., 2021), and resulted in growing demand for methods and technologies that enable rapid testing for antimicrobial susceptibility (Broddrick et al., 2020; Kaprou et al., 2021).

The rapid detection of pathogens and the administration of effective antibiotics are essential steps toward improving the prognosis of critically ill patients and minimizing hospital stays. Traditional microbiological detection techniques, including the gold standards of culture-, serum immunology-, and polymerase chain reaction (PCR)-based detection technologies, are each accompanied by obvious drawbacks. The lengthy time requirement for cultivation of atypical pathogens and unculturability of many microbes limit the timely identification of pathogens in most clinical microbiology laboratories. Similarly, serum immunological examination and PCR techniques can only detect a limited array of specific, well-characterized pathogens. Commercial multiplex PCR-based pathogen panels can also cover several, but not all known infection-causing pathogens and thus fail to detect many bacterial strains (Chen et al., 2020; Geng et al., 2021).

The emerging field of metagenomics has the potential to revolutionize pathogen detection in public health laboratories. with the development of not only the laboratory methods but also the bioinformatics improvements, metagenomic sequencing enables simultaneous and unbiased identification of all microorganisms in a clinical sample within 24–48 h of receipt, without *a priori* knowledge of their identities. This approach therefore offers several non-trivial advantages in determining the causative agents of conditional pathogen infections and mixed infections, and is also suitable for rare, novel, and atypical etiologies of complicated infectious diseases (Miller et al., 2013; Miao et al., 2018; Li et al., 2020). Previous reports have demonstrated the use of metagenomic next-generation sequencing for unbiased pathogen detection, producing results in shorter timeframes relevant to clinical diagnostics and public health. However, the development of rapid analytical methods based on the Illumina or Ion Torrent platforms are hampered by the long run time required to achieve sufficient sequencing depth for pathogen identification, since sequencing reads for these platforms are not allowed processing as they are generated so that the data is not available until whole sequencing run stopping (Greninger et al., 2015).

By contrast, nanopore sequencing, a third-generation sequencing technology, provides two key advantages over second-generation technologies, including longer reads and the capability of real-time sequence analysis (Cao et al., 2016; Broddrick et al., 2020), thus providing a viable avenue for fast and accurate pathogen identification as well as resistance gene detection (Taxt et al., 2020; Yang et al., 2020; Xu et al., 2021; Avershina et al., 2022). Previous studies have shown the use of nanopore sequencing in the detection of some clinical pathogens such as lower respiratory infections (Pendleton et al., 2017; Charalampous et al., 2019; Lewandowski et al., 2019), prosthetic joint infections (Sanderson et al., 2018), meningitis (Moon et al., 2017; Nakagawa et al., 2019), and for UTI diagnosis (Schmidt et al., 2017). As nanopore sequencing technology rapidly develops, numerous library preparation methods are updated and new protocols are established. Here, we compare three library preparation methods which are commonly used in studies of clinical pathogen diagnosis in order to determine the optimal detection method for UTIs. Furthermore, we demonstrate the feasibility and potential of metagenomic nanopore sequencing (mNPS) for diagnosis of unknown UTI pathogens.

## Materials and Methods

### Study Design

**FIGURE 1:**
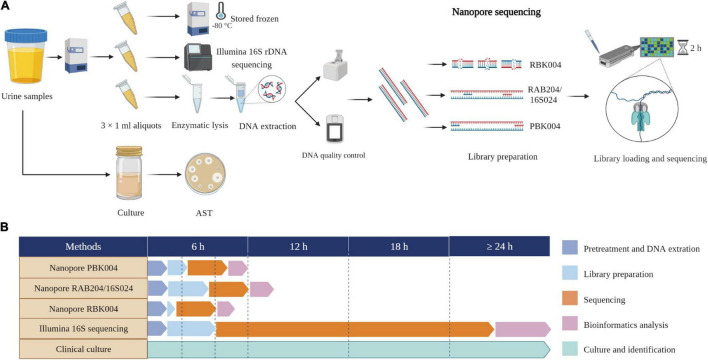
Study workflow and time comparison. **(A)** Schematic of mNPS assay workflow. One aliquot performed nanopore sequencing and the other two aliquots performed Illumina 16S rDNA sequencing and stored frozen, respectively. Clinical urine culture was performed by clinical doctors. **(B)** Timing for mNPS testing relative to culture and Illumina sequencing. The turnaround time for sample-to-detection of mNPS testing, defined here as the cumulative time taken for sample pretreatment and DNA extraction, library preparation incorporating beads clean-up, sequencing, bioinformatics analysis, pathogen and ARGs identification, was under 6 h, while Illumina sequencing took over 24 h and culture-based pathogen identification can take days to weeks. Created with BioRender.com.

We established a diagnostic platform for UTIs based on mNPS and subsequent data analysis. The workflow of our in-house mNPS platform includes DNA extraction, library preparation, sequencing, and pathogen identification (Figure 1).

In the first phase of our study, we assessed three methods of library preparation (rapid barcoding sequencing with RBK004, 16S sequencing with RAB204/16S024, and PCR barcoding sequencing with PBK004) using twenty culture-positive samples by comparing the consistency of mNPS results with culture-based results. Comparisons used the following criteria to evaluate library preparation methods: the sensitivity [referring to the positive criteria of sequencing established by Baldan et al. (2021) as follows: mono-species with largest number of reads, ≥ 10% difference from the next most abundant species] (Baldan et al., 2021), total reads number, the proportion of target reads and consistency in the detection of antibiotic resistance genes (ARGs) compared to clinical results. We then used the most comprehensive library preparation method to conduct mNPS testing on the remaining 56 samples.

### Sample Selection and Processing

We prospectively reviewed 76 cases of suspected acute or chronic infection between December 2020 and May 2021 at Dongfang Hospital in Beijing, China. Urine samples were collected *via* midstream clean catch for non-indwelled catheters patients and transurethral catheterization for indwelled patients, respectively. The urine remnants after standard-of-care clinical laboratory testing were then stored at −80°C before mNPS testing. The following criteria were used to determine inclusion in this study: no history of urological disorders and no prior clinical diagnosis of UTIs based on cell counts and cultures; with a few symptoms including urinary urgency, frequent urination, painful urination. A clinical diagnosis of UTI required to refer to the culture result and consider indicators including a white blood cell (WBC) count of > 10^7^/L, an Epithelial cell (EC) count of < 10^7^/L, fever, dysuria, frequency of urination, and urgency. WBC, EC, and red blood cell (RBC) counts were performed using phase-contrast microscopy. Urine cultures were performed on both MacConkey and blood agar plates, with incubation at 35°C for 16–18 h under aerobic conditions (Willner et al., 2014; Kumar et al., 2015). Demographic and baseline characteristics, clinical presentation, and laboratory findings of 76 patients were investigated for clinical diagnosis. Collection of surplus clinical samples was conducted under ethical approval by the Institutional Review Board (IRB) of the Beijing Dongfang Hospital (reference no. JDF-IRB-2020003101). All samples were obtained with the patient’s consent.

### Clinical Culture and Antibiotic Susceptibility Testing

Clinical microbiological analysis of urine samples was performed according to standards formulated by the Clinical and Laboratory Standards Institute (CLSI). Briefly, 10 μl of urine were inoculated onto blood agar plates (bioMérieux) and incubated for 16–24 h at 35 ± 2°C in aerobic conditions. If cultures were negative for colony formation, plates were incubated continually until 48 h. Bacterial counts were determined from 10^2^ to > 10^5^ CFU/ml. Bacterial identification and antibiotic susceptibility testing (AST) were performed separately with the VitekMS and VITECK2 Compact Systems (bioMérieux, SA), using the antimicrobial susceptibility test cards of the VITEK 2 AST-N334 and AST-GP67 Test Kits (bioMérieux, SA) for Gram-negative and Gram-positive organisms, respectively.

### Pre-treatment of Samples and DNA Extraction

Urine samples were removed from storage at −80°C and incubated at room temperature until completely thawed. Samples were then gently shaken until well-mixed, and 1 ml of each sample was aliquoted into 1.5 ml Eppendorf tubes (Eppendorf) and centrifuged at 20,000 × g for 5 min to enrich for bacteria. The resulting bacterial pellet was resuspended in 180 μl phosphate-buffered saline (PBS) with brief, gentle vortexing. Then, 5 μl of lytic enzyme solution (Qiagen) and 10 μl of MetaPolyzyme (Sigma Aldrich; reconstituted in 750 μl PBS) were added to the samples and mixed by pipetting. Mixed samples were incubated for 1 h at 37°C to lyse bacterial cells. DNA was extracted from each post-lysed sample using an IndiSpin Pathogen Kit (Indical Bioscience). Sterile deionized water was extracted alongside the specimens as a negative control. DNA concentrations were assessed using a Qubit 4.0 fluorometer with the dsDNA HS Assay kit (Thermo Fisher Scientific).

### MinION Library Preparation and Sequencing

All the samples included in this study were processed and sequenced regardless of DNA concentrations to provide an accurate representation of the data that would likely be obtained from metagenomic analysis of samples in clinical settings.

Library preparation for the MinION platform was performed according to the manufacturer’s protocols (see Supplementary Methods) for (1) 16S sequencing using a 16S Barcoding Kit (SQK-RAB204/SQK-16S024), (2) rapid sequencing with a Rapid Barcoding Kit (SQK-RBK004), and (3) PCR sequencing by PCR Barcoding Kit (SQK-PBK004), with the following minor alterations. The 16S PCR reaction was performed as per the manufacturer’s instructions. However, the number of PCR cycles was increased to 30. For PBK method, we used a 2 min extension time and 15 cycles. We selected 20 samples that were positive for clinical microbial culture and prepared libraries for each sample using all three of the above methods. The other samples were only sequenced using the PBK method.

MinION sequencing was performed using R9.4.1 flow cells (FLO-MIN106). A total of 75 μl of library DNA was loaded into the flow cell according to the manufacturer’s instructions. The MinION instrument was run for approximately 2 h. ONT MinKNOW GUI software (version 4.2.8) was used to collect raw sequencing data.

### MinION Data Bioinformatic Analysis

**FIGURE 2:**
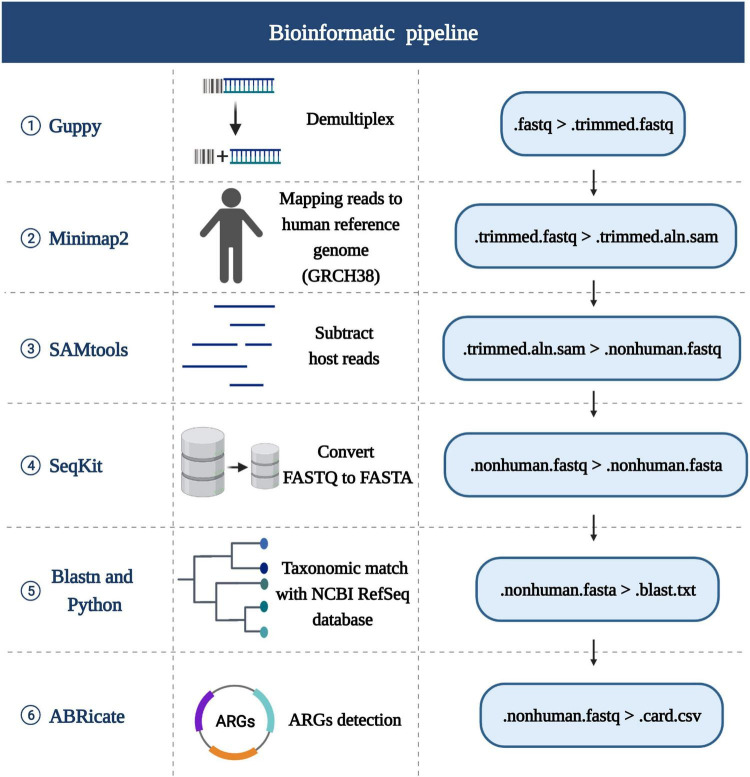
Schematic of the bioinformatics analysis pipeline. The first row lists the tools used for each stage, the second row lists the certain functionality of each step in the pipeline, the third row shows the detailed steps. Created with BioRender.com.

Figure 2 provides an overview of the bioinformatic workflow and the specific role of each tool in the process of MinION data analysis.

#### Bacterial Species Identification

**FIGURE 3:**
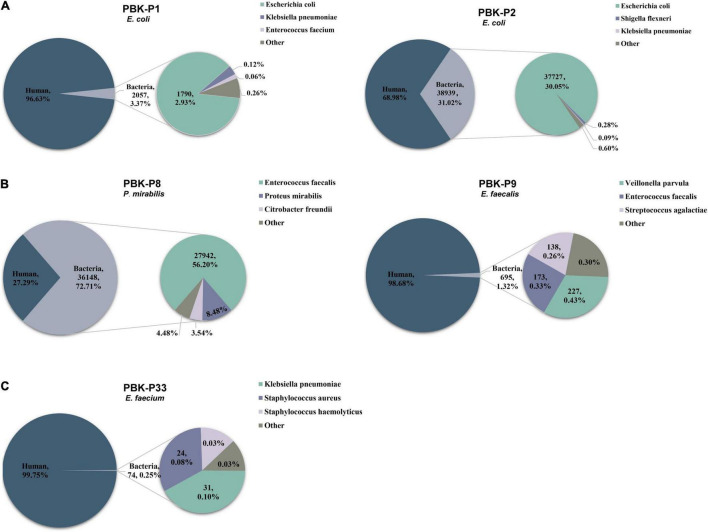
Pie charts demonstrating the taxonomic classification of reads for three different situations and showing the top 3 bacteria with percentage distributions of sequencing reads. Different colors indicate different species, the title indicates the method of library preparation, sample ID, and culture-based result. **(A)** Examples had one obviously dominant bacterial taxon. **(B)** Examples had two or more apparently predominant bacterial strains. **(C)** Examples with no obvious dominant bacteria and relatively few bacterial reads.

The sequencing reads were generated by MinIT with Guppy software (version 4.3.4, Oxford Nanopore) *via* real time base-calling. The barcodes and adapters were trimmed using Guppy with the command “guppy-barcoder.” Following demultiplexing, Minimap2 (version 2.17) (Li, 2018) was used to computationally subtract host reads, with the ‘‘-ax map-ont’’ setting, by aligning reads to the human reference genome (GRCH38).^1^ All remaining non-human reads were separated by SAMtools (version 1.7) (Li et al., 2009) and the FASTQ file outputs were converted to FASTA files with SeqKit (version 0.13.2) (Shen et al., 2016). FASTA reads were then mapped to the RefSeq bacterial database (containing 2328 bacterial genomes or scaffolds)^2^ by BLASTn (version 2.10.1). To make the results after BLASTn more intuitive, two python scripts were developed and performed, which are available in https://github.com/gitzl222/mNPS/, and the community profiles and abundance estimation graphs with information on the number and percentage of reads mapping to bacterial species are shown as a pie chart (see Figure 3 and Supplementary Figure 1). Criteria set for identification of bacterial pathogens by mNPS testing is listed below.

#### Criteria for a Positive Nanopore Sequencing Result

To minimize false-positive results from low-level urethral colonized flora, threshold criteria were established for pathogen detection. We defined an RPM (Reads Percent of Microbe) value referring to Gu et al. (2020), means the percentage of pathogen reads in total reads after demultiplexing. In order to determine the optimal threshold value for RPM and maximize the accuracy of pathogen detection, we plotted receiver operator characteristic (ROC) curve at varying RPM values that corresponded to mNPS analysis of the training set (35 samples), which used for evaluation of accuracy and determination of RPM threshold based on Youden’s Index. The ROC curve was plotted using Graphpad prism 8 software. Criteria for positive results of candidate pathogen detection met the following conditions: (1) ≥ 100 total pathogen reads identified, and (2) meet the optimal RPM threshold. After that, all pathogens which met positive Criteria of mNPS above were investigated in the PubMed database to determine whether they had been reported as probiotic like *Lactobacillus crispatus* (Stapleton et al., 2011) and if so, excluded it from pathogens list.

#### Antibiotic Resistance Gene Detection

Antibiotic resistance genes (ARGs) were identified by ABRicate (version 0.8)^3^ using the Comprehensive Antibiotic Research Database (CARD) (Jia et al., 2017; Hendriksen et al., 2019), with the input of non-human reads and the parameters set as follows: “–minid 80 –mincov 80 –csv” (Leggett et al., 2020; Sheka et al., 2021). This parameter currently reports resistance genes, acquired and chromosomal, but not resistance mutations/SNPs, with coverage and identity ≥ 80% (Charalampous et al., 2019). We identified, counted, and classified the numbers and types of ARGs in different pathogen species among 31 samples and visualized by R (version 4.0.4).

### Confirmatory Illumina 16S Sequencing

In cases of discrepancy between the results of mNPS and urine cultures, we sent 1 ml aliquots of the samples for Illumina 16S rDNA sequencing through an independent third-party company (LC Biotech, Hangzhou, China). Sequencing of the V3–V4 region of the 16S rDNA provided genus-based species distribution. The detailed methods are provided in Supplementary Methods.

### Initial Evaluation for Limits of Detection

To evaluate the limit of detection for our mNPS testing, we chose *Escherichia coli* and *Enterococcus faecium* as the representative species spiked into healthy donor negative urine samples with diluted concentrations of 10^3^, 10^4^, and 10^5^CFU/ml. Then we subjected these samples to our described mNPS testing by PBK method.

### Statistical Analysis

Statistical analysis to assess test accuracy used a two-tailed Mann-Whitney *U*-test at a significance threshold of *P* = 0.05 to evaluate if there were systematic differences in total reads, host reads, and bacterial reads between culture-positive and culture-negative samples. A *P*-value < 0.05 indicated significant differences. The sensitivity and specificity of tests were calculated based on the mNPS criteria for pathogen detection. All the above statistical analyses were performed with Graphpad 8.

We used urine culture as a clinical gold standard, and Illumina 16S rDNA sequencing as the confirmatory testing. The specific scoring algorithm is outlined as follows (see Supplementary Table 1): based on the clinical gold standard, for the culture-positive samples, true positives or false negatives were scored for each microorganism detected or not detected by mNPS positive criteria, respectively; for the culture-negative samples, true negatives were scored if no microorganism was detected by mNPS, otherwise, a false positive was scored. When multiple microorganisms were detected as positive by mNPS in one culture-negative sample, we counted all these false-positive microorganisms as one false positive result overall.

## Results

### Patient Characteristics and Conventional Identification

Demographic features of the 76 patients in this study are provided in Table 1. For culture-based identification, among the 76 samples, 45 (59.2%) were culture-positive, including 17 *Escherichia coli* (37.8%), 12 *Enterococcus* spp. (26.7%), 4 *Klebsiella pneumoniae* (8.9%), 3 *Corynebacterium* spp. (6.7%), 2 *Proteus mirabilis* (4.4%), 2 *Staphylococcus epidermidis* (4.4%), 2 *Staphylococcus haemolyticus* (4.4%), 1 *Pseudomonas aeruginosa* (2.2%), 1 *Enterobacter hormaechei* (2.2%), and 1 *Morganella morganii* (2.2%). The remaining 31 (40.8%) were culture-negative. We performed AST analysis of the 43 culture-positive samples (expect 2 *Corynebacterium striatum* samples have no AST result) and found that 31 (72.1%) were resistant to fluoroquinolone, 24 (55.8%) to penicillin, 16 (37.2%) to sulfonamide, 14 (32.6%) to cephalosporin, aminoglycoside and macrolide, 10 (23.3%) to nitrofurantoin, 9 (20.9%) to monobactam, 5 (11.6%) to tetracycline, 2 (4.7%) to carbapenem, cephamycin and nitroimidazole, and 1 (2.3%) to lincosamide, streptogramin, and glycopeptide. Detailed results of AST are shown in Supplementary Table 2.

**TABLE 1:** Characteristics of patients and laboratory findings.

Patient demographics (*n* = 76)			
**Age (Years)**		**Gender, no. (%)**	
Mean	70	Female	40 (52.6)
Range	30–97	Male	36 (47.4)
**Comorbidities, no. (%)**		**Days hospitalized**	
Chronic kidney disease	4 (5.3)	Median	20
Active malignancy	24 (31.6)	Range	0–81
Arthritis	3 (3.9)		
Diabetes	4 (5.3)		
Underlying infectious syndromes	8 (10.5)		
Other	33 (43.4)		

**Laboratory findings**			

**PCT, ng/ml, no. (%) (reference range, < 0.05)**		**CRP, mg/L, no. (%) (reference range, < 10)**	
Unknown	24 (31.6)	Unknown	10 (13.2)
<0.05	10 (13.2)	<10	20 (26.3)
0.05–0.5	26 (34.2)	10–90	24 (31.6)
>0.5	16 (21.1)	>90	22 (28.9)
**WBCcount,/μl, no. (%) (reference range, 0–30)**		**Percentage of neutrophils, no. (%) (reference range, 40–75)**	
Unknown	1 (1.3)	Unknown	5 (6.6)
0–30	31 (40.8)	<40	3 (3.9)
>30	44 (57.9)	40–75	30 (39.5)
Range	1–10,000	>75	38 (50.0)
**Organism cultured, no. (%)**			
*Enterobacter* spp.	22 (28.9)		
*Enterococcus* spp.	12 (15.8)		
*Staphylococcus* spp.	4 (5.3)		
Negative	31 (40.8)		
Other	7 (9.2)		

The mean patient age was 70 years and 52.6% were female. Among the 76 patients, 71 (93.4%) were hospitalized and the median duration of hospitalization was 20 days. Information on the WBC counts, C-reactive protein, and procalcitonin inflammation indicators of all patients is also listed in Table 1.

### Comparison of Performance Among the Three Library Preparation Methods

In order to select the most suitable library preparation method for our mNPS platform, we compared the detection results of the three methods, using clinical urine culture as the gold standard. We found that 16S sequencing results were concordant with culture-based identification in 16 (80%) of the 20 samples. Notably, the rapid barcode sequencing results were concordant with only 11 (55%) of the 20 of the culture assays, while the remaining 9 samples showed disagreement in their identification or were negative for pathogenic taxa. Sequencing results from the PBK method were concordant with the culture results of 16 (80%) samples, although one could only identify bacteria to the genus level (*Corynebacterium* spp.). The detailed results are listed in Supplementary Table 3. These results indicate that library preparation by the PBK method could provide higher consistency with culture-based assays than that of the RBK method. In addition, the PBK method enabled deeper exploration of the genomic information relevant to drug resistance genes compared to 16S method. Taken together, these findings indicate that library preparation by PBK could provide the most reliable and informative results for mNPS-based pathogen detection in the full set of culture-positive samples.

### Comparison of Reads Distribution Between Culture-Positive and Culture-Negative Samples and Taxonomic Classification by Metagenomic Nanopore Sequencing With PCR Barcoding Kit Method

Based on our above results, we then used the PBK method to generate libraries and performed sequencing for the remaining set of 56 clinical urine samples. We compared the total reads, percentage of host reads, and bacterial reads in the culture-positive and culture-negative samples, respectively (see Table 2 for medians, IQR, and range, see Supplementary Table 4 for raw data). We found that the total read counts showed no significant difference between the culture-positive and culture-negative samples (*P* = 0.7928), whereas the proportion of host reads was significantly lower (*P* < 0.0001) and the proportion of bacterial reads was significantly higher (*P* < 0.0001) in culture-positive samples compared with those of culture-negative samples. We examined the distribution of host reads and the top 3 most abundant bacterial reads (Figure 3) and found that 73.3% of samples had one obviously dominant bacterial taxon (e.g., P1 and P2, Figure 3A), while 11.1% of samples had two or more apparently predominant bacterial strains (e.g., P8 and P9, Figure 3B). There were also 8.9% of samples with no obvious dominant bacteria and relatively few bacterial reads (e.g., P33, Figure 3C).

**TABLE 2:** Comparison of reads information between culture-positive and culture-negative samples.

		Total reads*^a^*	Host reads	Proportion*^b^*	Bacterial reads	Proportion*^c^*	Length (bp) of bacterial reads
**Culture-positive**	**Median**	52,517	39,566	68.98%	15,625	31.02%	766
	**IQR**	36,737–75,443	13,162–46,976	33.39–92.11%	2,273–27,670	7.89–66.61%	592.6–994.4
	**Range**	6,232–152,759	1,005–147,819	3.36–99.75%	74–96,446	0.25–96.64%	319.3–1525.4
**Culture-negative**	**Median**	57,347	57,223	99.78%	110	0.22%	472.7
	**IQR**	30077.5–89887.5	29933.5–89363	99.6–99.86%	47.5–199	0.14–0.4%	428.4–580.6
	**Range**	2,065–174,474	2,018–173,531	97.72–99.95%	9–1,832	0.12–2.28%	306.9–1209.6

*^a^Two-tailed Mann-Whitney U-test indicates no significant difference at P = 0.7928.*

*^b^Two-tailed Mann-Whitney U-test indicates significant difference at P < 0.0001.*

*^c^Two-tailed Mann-Whitney U-test indicates significant difference at P < 0.0001.*

### Test Accuracy

**FIGURE 4:**
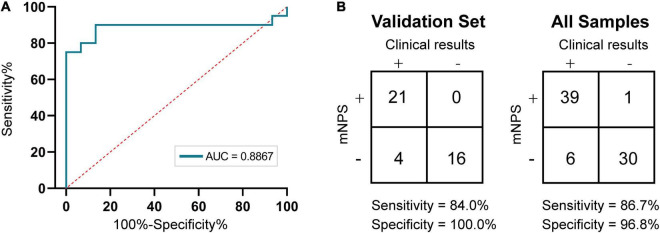
Accuracy of mNPS testing. **(A)** ROC curve of nanopore sequencing of training set based on culture. Plotted are mNPS test sensitivities and specificities, relative to the clinical urine culture, at RPM threshold values ranging from 0.04 to 91.24. **(B)** Contingency table for the training set (*n* = 35 samples) and validation set (*n* = 41 samples) of mNPS, respectively. The scoring system for determination of positive and negative results is listed in Supplementary Table 1.

Evaluation of test accuracy focused on the performance of mNPS testing in the identification of causal UTI pathogens relative to that of culture-based diagnostics. After evaluation of library preparation methods, we divided all the 76 samples into a training set (*n* = 35, including 20 culture-positive samples and 15 culture-negative samples) and a validation set (*n* = 41, including 25 culture-positive samples and 16 culture-negative samples) randomly. ROC curve was generated for the training set based on the results of clinical culture using the RPM metric (Figure 4).

Then, at the optimal Youden’s index derived from the training set ROC curve, the RPM threshold was set to 0.3065 with an AUC value of 0.8867. After meeting the precondition of ≥ 100 total pathogen reads, a minimum threshold of 0.3065 RPM was designated for reporting the positive or negative detection of a bacterial taxon (RPM ≥ 0.3065 or < 0.3065 for positive or negative, respectively). We then investigated the mNPS result of spiked-in samples based on positive criteria and found that all the species of ≥ 10^4^CFU/ml were successfully detected as positives. However, the remaining two samples with 10^3^CFU/ml spiked-in species were only detected as positive in one, with an RPM = 1.4 (see Supplementary Table 5). Next, we used the results of clinical culture to establish the sensitivity and specificity of bacterial detection by mNPS testing for the validation set and all the sample set, respectively. The results showed that mNPS-based diagnostic detection had 84.0% (95% CI, 64.7–94.2%) sensitivity and 100% (95% CI, 77.3–100%) specificity for the validation set and 86.7% (95% CI, 73.5–94.1%) sensitivity and 96.8% (95% CI, 82.4–99.9%) specificity for all the sample set compared to the standard clinical culture method.

Based on criteria for positive detection, sequencing results were in agreement with culture-based detection in 39 of 45 culture-positive samples (86.7%). The six remaining mNPS-negative samples were therefore considered false negatives (see Supplementary Table 1). In the case of P26, both clinical culture and mNPS found the presence of bacterial pathogen(s), but disagreed on the pathogen identification, with mNPS indicating the presence of dual-dominant pathogens *K. pneumoniae* and *Lactobacillus delbrueckii* and culture-based identification indicating the presence of *P. mirabilis*. To resolve this discrepancy, we conducted Illumina 16S rDNA sequencing and found that sample P26 was indeed positive for the two strains identified by mNPS, which supported the credibility of the mNPS identification method. In the case of sample P33, insufficient reads were obtained by mNPS to meet the criteria for a positive detection, whereas culture methods indicated the presence of *E. faecium*. Illumina 16S rDNA sequencing revealed the pathogenic agent as *Enterobacter* spp., which was also inconsistent with culture results (see Supplementary Table 1). In the case of sample P13 and P43, the culture results suggested that these two samples have bacterial counts of 10^3^CFU/ml and with our mNPS method it is still somewhat difficult to detect such low biomass samples, which is also in line with the result of spiked specimens (see Supplementary Table 5).

Among the 31 culture-negative samples, only one (N14) met the threshold for an mNPS-positive ID of *E. coli*, which was undetected by culture-based testing and therefore designated as a false positive. Among the other culture-negative samples, none met the mNPS threshold criteria for positive detection and were therefore considered true negatives (see Supplementary Table 1).

### Detection of Antibiotic Resistance Genes by Metagenomic Nanopore Sequencing

**FIGURE 5:**
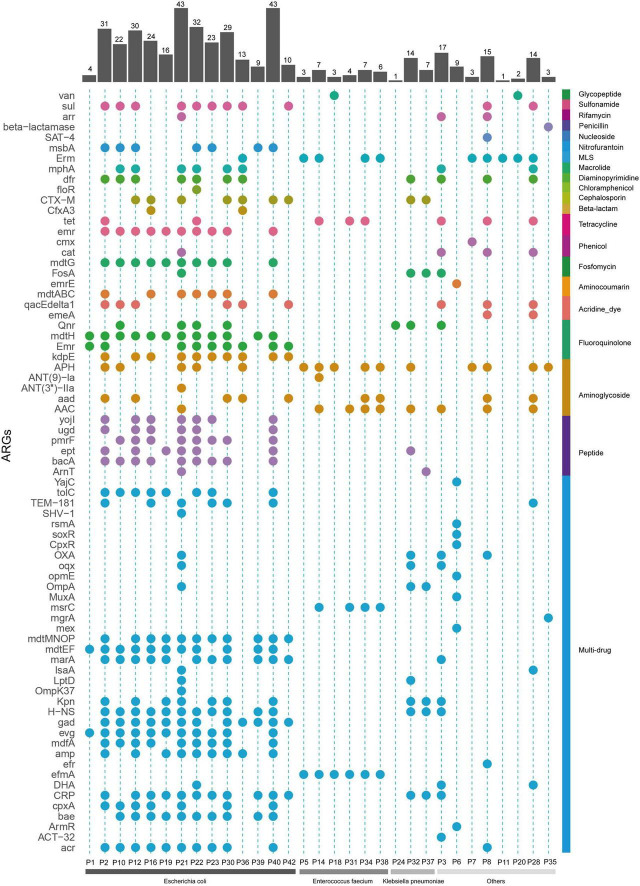
Resistance genes profile of all the mNPS-based positive samples. Colors are to aid visual interpretation. Heatmap strip at the right with different colors represent different types of antimicrobial class. Heatmap strip at the bottom represents different pathogen species. Bar chart indicates the number of ARGs per sample.

Following pathogen detection, we next explored the sequencing data to screen for antibiotic resistance genes (ARGs) in the 39 mNPS-positive samples. This analysis revealed a total of 73 ARGs across 31 samples, with 8 samples showing no mNPS reads that mapped to known ARGs. In these 31 samples, the largest number of ARGs per sample mapped to *E. coli* (Figure 5). Among the 73 identified ARGs, most were efflux components or β-lactamase genes, several of which conferred multidrug resistance. The abundance of ARGs in each sample and the corresponding types of antibacterial agents are shown in Figure 5. Multidrug resistance-related genes accounted for the largest proportion. Notably, the number of ARGs in output data was positively correlated with sequencing depth, since limited reads in some samples consequently also limited the number of detectable ARGs (e.g., P1 and P24). The AST results indicated that β-lactam, fluoroquinolone, and cotrimoxazole resistance appeared at the highest frequencies. We then compared the AGRs output with clinical AST results to investigate the concordance between the both (excluded 2 samples without AST result). The two methods produced fully concordant results in 6 (20.7%) samples and partially consistent results in the remaining 23 (79.3%) samples, and nitrofurantoin was the most frequently missed in ARG screening of the mNPS data. In addition, we found that all ESBL phenotypes conferred resistance to third generation cephalosporins due to the presence of *blaCTX-M* genes and the majority of fluoroquinolone resistance was due to *CRP*, *emrH*, and/or *gadX* genes. We also detected the *vanA* gene which is associated with vancomycin resistance in sample P18 and AST results indicated that it was indeed a vancomycin resistant strain. Other ARGs conferred resistance to untested and non-clinically relevant antibiotics (e.g., acridine_dye and rhodamine).

## Discussion

Here, we developed and analytically validated a rapid metagenomic nanopore sequencing (mNPS) diagnostic assay for unbiased identification of UTI bacterial pathogens and antibiotic resistance genes within 6 h of sample receipt, providing obvious advantages in turnaround time over that of Illumina-based sequencing (Greninger et al., 2015). In addition, we evaluated three different library-preparation methods using twenty culture positive samples and determined that the PBK method resulted in the highest percentage of positive diagnosis that agreed with clinical urine culture. Collectively, our results showed that PBK library preparation not only provides the highest sensitivity among these three methods, but can also facilitate the identification of ARGs in samples, which is unavailable through other library preparation methods, thus indicating that PBK allowed the most comprehensive analysis of metagenomic data.

Mechanical lysis has been widely adopted for DNA extraction in both Gram-positive and Gram-negative bacteria within complex matrices. However, DNA obtained through bead beating is largely too sheared to produce long reads, potentially reducing the accuracy of pathogen identification (Chiu and Miller, 2019). In consideration of these issues, we modified the DNA extraction protocol to use a combination of lytic enzyme solution and MetaPolyzyme for effective lysis of a range of microbes with minimal shearing, as described by Maghini et al. (2021) demonstrated the capability of relatively consistent lysis from both Gram-positive and Gram-negative organism compared to the mechanical lysis and the advantage in scenarios with limited input sample volume.

We considered several possibilities that the sensitivity of RBK methods was slightly lower. First, the initial DNA concentration was too low to meet the high baseline sample input (400 ng) of the standard process in RBK method. As a result, the output reads were insufficient for identification. Second, the shorter sequences (average 235.6 bp) by RBK increased the probability of non-specific alignment and led to incorrect alignment output. While the PBK method with no fragmentation have longer reads (average 778 bp) and higher blasting accuracy (Supplementary Figure 2). The high sequencing error of R9.4 nanopore is third factor for that can probably result in some misdiagnoses. Notably, future improvement of nanopore technology on read accuracy can be expected to solve this problem (Wang et al., 2021).

Among all patients included in this study, the most frequently appearing UTI pathogens were *Enterobacter* spp., among them, *E. coli* was found to be a major causative agent of UTIs in this work, which has been found in other studies to harbor genes conferring multiple drug-resistance (Schwartz et al., 2011; O’Brien et al., 2018; Finton et al., 2020). *Enterococcus* spp. were another frequently appearing group of UTI pathogens which are also representative of MDR bacteria and have been described as an important threat in the World Health Organization global pathogen priority list (Rello et al., 2020). The mNPS method shown here can provide fast and accurate guidance for targeted therapeutic of mNPS-positive pathogens in UTI patients.

Previously published studies have used mNPS for pathogen detection in bloodstream infections, meningitis, and orthopedic infections (Sanderson et al., 2018; Nakagawa et al., 2019; Sakai et al., 2019), as well as for pathogen detection in UTIs (Schmidt et al., 2017). However, the reported test for UTIs used heavily infected urine samples with ≥ 10^7^CFU/ml, while we successfully detected pathogens with a considerably lower biomass of 10^3^–10^4^ CFU/ml in some clinical samples and spiked samples, such as samples P15, P27, P44 and L2, L5, L6 (see Supplementary Table 5). In addition, the method described here requires only 2 h of sequencing time compared to ≥ 24 h sequencing time in the previous work. Although the sequencing reaction was performed for 2 h in this study, we found that, in the vast majority of cases, we were able to obtain sufficient reads from the MinION to identify the causative pathogen(s) in less than 30 min, as obtained for samples P2 and P3. We also perform count analysis of the sequencing reads according to its generated time for six samples and found that the percentages of reads aligned to the pathogen were equivalent regardless of the duration of the sequencing (see Supplementary Table 6), which is also in line with the results of a published study (Moon et al., 2019).

Several studies have indicated that the urine of healthy people is not sterile and can include a relatively high number of urethral colonized flora. For example, the *L. delbrueckii* is a commensal species of bacteria that has been reported to potentially protect UT against invasion by uropathogens, which exclude the possibility for samples such as N19 to be determined as pathogen-positives by mNPS testing in this study (Siddiqui et al., 2011; Moustafa et al., 2018; Neugent et al., 2020). This finding suggests that shifts in the prevalence of natural flora in the urethra could result in the emergence of potential pathogens, and ultimately the onset of UTIs. Our sequencing results also suggested that several samples contained multi-bacterial infections, such as P8, P9, and P20, although clinical culture only identified a single bacterial pathogen in P8 and P9. Illumina 16S rDNA sequencing confirmed the positive results obtained by MinION sequencing indicating multi-bacterial infection. These findings highlight the major advantages of mNPS in the diagnosis of multi-bacterial infections.

The application of genome sequencing to predict drug resistance in pathogens has been well-established for many years (Hendriksen et al., 2019; Aytan-Aktug et al., 2021). However, nanopore sequencing has the potential to obtain more accurate prediction of antibiotic resistance due to the long read lengths, although the ability to detect ARGs may be limited in cases of low pathogen titer within the total microbiome. This issue can be potentially resolved by increasing sequencing depth. However, resistance mechanisms may also emerge through point mutations, structural variations that induce changes in gene expression, and posttranslational modifications, among others. Consequently, the workflows of our methods can be used to identify resistance mechanisms derived from the acquisition of specific resistance genes such as *dfrA/B, sul1/2*, or *lsaA*, etc., but cannot comprehensively detect all potential resistance mechanisms.

We found a larger number of ARGs in the data obtained from mNPS analysis of urine samples than that provided by AST results. This result was unsurprising, since urine contain high number of species originating from the urethral microbiome as we described above and additionally, the presence of resistance genes does not always correlate with phenotypic resistance (Siddiqui et al., 2011; Moustafa et al., 2018). These issues could lead to potential overestimation of the occurrence of resistance in patient samples and possibly treatment with broader-spectrum antibiotics that is unnecessary (Hasman et al., 2014). However, filtering low coverage genes resulted in removal of almost all resistance genes that did not correspond with AST, regardless of the fact that direct sequencing could lead to slight overestimation of drug resistance among potential pathogens. It is noteworthy that this procedure did not miss any genes, compared to clinical AST.

It is easy to envision that our mNPS method described here can be widely adopted in clinical settings, but is currently subject to high material costs, especially the PCR barcoding method, with additional and more expensive reagents to prepare the specific DNA end compared with some classic methods like rapid barcoding method and 16S barcoding method. To improve cost-effectiveness, we pooled the respective libraries and saved reagents by performing multiplex sequencing. Additionally, we also washed the flow cell with the Flow Cell Wash Kit (EXP-WSH004) after the sequencing reaction ended so that it could be reused. As a result, with 6 libraries pooled each run and reused the flow cell 4–6 times, the cost could be down to ∼$92 from ∼$1598 for library preparation and sequencing of each sample, greatly increasing the adaption of mNPS for clinical diagnosis.

Limitations of our study include the following: First, confirmatory Illumina 16S rDNA sequencing was not performed on all samples, and further validation by a third method is needed. Second, clinical samples had high percentage of host DNA and varying depths of sequencing for pathogens, which may have contributed to some of the false-negative results and limited ARG outputs. Third, sequencing of pathogenic bacteria isolates in urine samples was not performed, therefore limiting the ability to confirm presence of ARGs by deep sequencing of purified isolates. Forth, we did not test other alternative classifiers other than BLASTn, which may have limited the potential for reaching a much higher speed of mNPS testing. Fifth, though we tried our best to avoid shearing of DNA when performed bacterial DNA extraction from urine samples, the DNA length seems still short. Thus, further improvement of the protocol for DNA extraction in our future work may be better. Finally, the sensitivity and specificity of mNPS testing are overestimated given that the testing and validation samples are not truly independent and the small sample size. Therefore, further evaluation of test sensitivity and specificity for mNPS is needed based on larger cohort of samples in the following study.

In conclusion, we determined the pathogen content of UTI samples by the PBK method and demonstrate its diagnostic feasibility. Our in-house platform mNPS shows strong potential for clinical adaptation. A turnaround time of less than 6 h is feasible with mNPS testing and may be highly effective for diagnosing infectious diseases for which a rapid response and timely diagnosis are urgently needed. We have also demonstrated the ability to obtain data showing the prevalence of ARGs by direct sequencing of clinical urine samples. Future work will focus on the adaptability of this method for samples of other types of infection. Our results show that mNPS-based diagnostic methods can serve as powerful clinical tools for therapeutic management of UTIs and other infectious diseases.

## Data Availability Statement

The MinION sequence reads and Illumina sequence reads can be accessed at the NCBI Sequence Read Archives under BioProject accession number PRJNA751627.

## Ethics Statement

The studies involving human participants were reviewed and approved by the Institutional Review Board (IRB) of the Beijing Dongfang Hospital (reference no. JDF-IRB-2020003101). The patients/participants provided their written informed consent to participate in this study.

## Author Contributions

PL, WH, YJ, and LZ conceived and designed the study. LZ, SZ, YW, and TC carried out the experimental work and analyzed the data. LZ and WH conceptualized the experimental methods, performed bioinformatics, and wrote the original draft of the manuscript. PL, WH, and YJ participated in the review and editing of the manuscript. All authors participated in interpreting the results, read and approved the final version of this manuscript.

## Conflict of Interest

The authors declare that the research was conducted in the absence of any commercial or financial relationships that could be construed as a potential conflict of interest.

## Publisher’s Note

All claims expressed in this article are solely those of the authors and do not necessarily represent those of their affiliated organizations, or those of the publisher, the editors and the reviewers. Any product that may be evaluated in this article, or claim that may be made by its manufacturer, is not guaranteed or endorsed by the publisher.

## Footnotes

1
https://ftp.ncbi.nlm.nih.gov/genomes/all/GCF/000/001/405/GCF_000001405.39_GRCh38.p13/

2
https://ftp.ncbi.nlm.nih.gov/genomes/refseq/

3
https://github.com/tseemann/abricate
