# Supplementary material for: Rapid Detection of Bacterial Pathogens and Antimicrobial Resistance Genes in Clinical Urine Samples With Urinary Tract Infection by Metagenomic Nanopore Sequencing
Source: Front Microbiol. 2022 May 17;13:858777. doi: 10.3389/fmicb.2022.858777 (PMC9152355; doi:10.3389/fmicb.2022.858777)
Supplement: Supplementary file 7 [file Presentation_1.PDF]

Confirmatory Illumina 16S sequencing (Served by LC Biotech, Hangzhou, China)

DNA extraction

DNA of all the samples were extracted using OMEGA Stool DNA Kit according to the manufacturer’s protocol.

Construction of target fragment library and sequencing

The PCR primer was designed against the conserved region to target the variable region of the 16S rDNA gene. After 35 cycles of PCR, sequencing adapters and barcodes were added for amplification. PCR amplification products were detected by 1.5% agarose gel electrophoresis. The target fragments were recovered using the AxyPrep PCR Cleanup Kit. The PCR product was further purified using the Quant-iT PicoGreen dsDNA Assay Kit. The library was quantified on the Promega QuantiFluor fluorescence quantification system. The pooled library was loaded on Illumina platform using a paired-end sequencing protocol (2×250 bp).

Bioinformatics pipeline

Paired-end reads was assigned to samples based on their unique barcode and truncated by cutting off the barcode and primer sequence. Paired-end reads were merged using FLASH (v1.2.8). Quality filtering on the raw reads were performed under specific filtering conditions to obtain the high-quality clean tags according to the fqtrim (v0.94). Chimeric sequences were filtered using Vsearch software (v2.3.4). The sequence alignment of species annotation was performed by BLASTn, and the alignment database was SILVA and NT-16S.

Operating Procedure of SQK-RBK004

1. In a 0.2 mL thin-walled PCR tube, mix the following:

Component	Volume
Near 400 ng template DNA	7.5 µL
Fragmentation Mix RB01-12 (one for each sample)	2.5 µL
**Total**	**10 µL**

2. Mix gently by flicking the tube, and spin down.
3. Incubate the tube at 30 °C for 1 minute and then at 80 °C for 1 minute. Briefly put the tube on ice to cool it down.
4. Pool all barcoded samples (no more than 8 samples), noting the total volume.
5. Preform Agencourt AMPure XP beads purification with 1:1 volume beads and elute with 10µL 10 mM Tris-HCl (pH 8.0 with 50mM NaCl).
6. Perform the rapid adapter (RAP) ligation and priming and loading the SpotON flow cell according to the universal protocol.
7. Wash the flow cell with SQK-WSH004 kit according to the universal washing protocol.

Operating Procedure of SQK-RAB204

1. In a Thaw the 16S Barcodes at RT, mix by pipetting up and down, and spin down briefly. Keep the barcodes on ice until ready to use.
2. Prepare the DNA in Nuclease-free water.
   1) Transfer 10 ng genomic DNA into a DNA LoBind tube.
   2) Adjust the volume to 10 µL with Nuclease-free water.
   3) Mix thoroughly by flicking the tube, to avoid unwanted shearing.
   4) Spin down briefly in a microfuge.
3. In a 0.2 mL thin-walled PCR tube, mix the following:

**Component**	**Volume**
Near 10 ng template DNA	10 µL
LongAmp Taq 2X master mix	25 µL
16S Barcode, at 10 µM	1 µL
Nuclease-free water	14 µL
**Total**	**50 µL**

4. Mix gently by flicking the tube, and spin down.
5. Amplify using the following cycling conditions:
   1) Initial denaturation 1 min @ 95 °C (1 cycle)
   2) Denaturation 20 secs @ 95 °C (30 cycles)
   3) Annealing 30 secs @ 55 °C (30 cycles)
   4) Extension 2 mins @ 65 °C (30 cycles)
   5) Final extension 5 mins @ 65 °C (1 cycle)
   6) Hold @ 4 °C
6. Transfer the sample to a clean 1.5 mL Eppendorf DNA LoBind tube.
7. Perform Agencourt AMPure XP beads purification with 30 µL beads and elute with 10µL 10 mM Tris-HCl (pH 8.0 with 50mM NaCl).
8. Pool all barcoded libraries in the desired ratios to a total of 50-100 fmoles in 10 µL of 10 mM Tris-HCl (pH 8.0 with 50 mM NaCl).
9. Perform the rapid adapter (RAP) ligation and priming and loading the SpotON flow cell according to the universal protocol.
10. Wash the flow cell with SQK-WSH004 kit according to the universal washing protocol.

Operating Procedure of SQK-16S024

1. Take one 96-well plate containing 16S barcodes. Break one set of barcodes (1-24, or as desired) away from the plate and return the rest to storage.
2. Thaw the desired barcodes, make sure the liquid is at the bottom of the tubes, and place on ice.
3. Thaw the LongAmp Hot Start Taq 2X Master Mix, spin down briefly, mix well by pipetting and place on ice.
4. Prepare the DNA in Nuclease-free water.
   1) Transfer 10 ng genomic DNA into a DNA LoBind tube.
   2) Adjust the volume to 10 μL with Nuclease-free water.
   3) Mix thoroughly by flicking the tube, to avoid unwanted shearing.
   4) Spin down briefly in a microfuge.
5. For each sample to be tested, prepare the following mixture in separate 0.2 mL thin-walled PCR tubes.

**Component**	**Volume**
Near 10 ng template DNA	10 μL
LongAmp Hot Start Taq 2X Master Mix	25 μL
Nuclease-free water	5 μL
**Total**	**40 μL**

6. Using clean pipette tips, carefully pierce the foil surface of the required barcodes. Use a new tip for each barcode to avoid cross-contamination. Make a note of which barcode numbers will be run for each sample.
7. Using a multichannel pipette, mix the 16S barcodes by pipetting up and down 10 times. Transfer 10 μL of each 16S Barcode into respective sample-containing tubes.
8. Mix thoroughly by pipetting up and down ten times.
9. Amplify using the following cycling conditions:
   1) Initial denaturation 1 min @ 95 °C (1 cycle)
   2) Denaturation 20 secs @ 95 °C (30 cycles)
   3) Annealing 30 secs @ 55 °C (30 cycles)
   4) Extension 2 mins @ 65 °C (30 cycles)
   5) Final extension 5 mins @ 65 °C (1 cycle)
   6) Hold @ 4 °C
10. Transfer the sample to a clean 1.5 mL Eppendorf DNA LoBind tube.
11. Perform Agencourt AMPure XP beads purification with 30 μL beads and elute with 10μL 10 mM Tris-HCl (pH 8.0 with 50mM NaCl).
12. Pool all barcoded libraries in the desired ratios to a total of 50-100 fmoles in 10 μL of 10 mM Tris-HCl (pH 8.0 with 50 mM NaCl).
13. Perform the rapid adapter (RAP) ligation and priming and loading the SpotON flow

cell according to the universal protocol.

14. Wash the flow cell with SQK-WSH004 kit according to the universal washing protocol.

Operating Procedure of SQK-PBK004

1. Prepare the NEBNext Ultra II End Repair/dA-Tailing Module reagents according to the manufacturer's instructions, and place on ice.
2. Mix the following reagents in a 0.2 mL thin-walled PCR tube:

**Component**	**Volume**
Near 100 ng template DNA	50 μL
Ultra II End-prep reaction buffer	7 μL
Ultra II End-prep enzyme mix	3 μL
**Total**	**60 μL**

3. Mix well by gently pipetting the entire volume within the tube up and down 10 times.
4. Using a thermal cycler, incubate at 20°C for 5 mins and 65°C for 5 mins.
5. Resuspend the AMPure XP beads by vortexing.
6. Transfer the sample to a clean 1.5 mL Eppendorf DNA LoBind tube.
7. Perform Agencourt AMPure XP beads purification with 60 μL beads and elute with 16 μL Nuclease-free water.
8. Thaw the Blunt/TA Ligase Master Mix, spin down and mix by pipetting the entire volume within the tube up and down 10 times. Check for any precipitate (if any is visible, continue to mix) and place on ice.
9. Thaw the Barcode Adapter (BCA), spin down and mix by pipetting the entire volume within the tube up and down 10 times. Place on ice.
10. Add the reagents in the order given below, mixing by flicking the tube between each sequential addition:

**Component**	**Volume**
End-prepped DNA	15 μL
Barcode Adapters (BCA)	10 μL
Blunt/TA Ligase Master Mix	25 μL
**Total**	**50 μL**

11. Mix well by gently pipetting the entire volume within the tube up and down 10 times.
12. Incubate the reaction for 10 minutes at RT.
13. Perform Agencourt AMPure XP beads purification with 30 μL beads and elute with 25 μl Nuclease-free water.
14. Quantify 1 μL of adapted DNA using a Qubit fluorometer.
15. Calculate how much DNA to take forward into the PCR step for a final DNA concentration of 0.2 ng/μL in a 50 μL reaction.
16. Thaw the LongAmp® Hot Start Taq 2X Master Mix at RT, spin down and mix by pipetting the entire volume within the tube up and down 10 times. Place on ice.

17. Thaw the required Barcode Primers (BP01-12) at RT, spin down and mix by pipetting the entire volume within the tube up and down 10 times. Place on ice.
18. Set up the adapted DNA PCR as follows:

**Component**	**Volume**
Adapter ligated DNA	diluted x µL 0.2 ng/µL
Barcode Primers (BP01-12, at 10 µM)	1 µL
LongAmp® Hot Start Taq 2x Master Mix	25 µL
Nuclease-free water	24-x µL
**Total**	**50 µL**

19. Mix well by gently pipetting the entire volume within the tube up and down 10 times.
20. Amplify using the following cycling conditions:
    1) Initial denaturation 3 mins @ 95 °C (1 cycle)
    2) Denaturation 15 secs @ 95 °C (15 cycles)
    3) Annealing 15 secs @ 56 °C (15 cycles)
    4) Extension 2 min @ 65 °C (15 cycles)
    5) Final extension 6 mins @ 65 °C (1 cycle)
    6) Hold @ 4 °C
21. Perform Agencourt AMPure XP beads purification with 50 µL beads and elute with 10µL 10 mM Tris-HCl (pH 8.0 with 50mM NaCl).
22. Pool all barcoded libraries in the desired ratios to a total of 50-100 fmoles in 10 µL of 10 mM Tris-HCl (pH 8.0 with 50 mM NaCl).
23. Perform the rapid adapter (RAP) ligation and priming and loading the SpotON flow cell according to the universal protocol.
24. Wash the flow cell with SQK-WSH004 kit according to the universal washing protocol.

Agencourt AMPure XP beads purification (Universal)

1. Resuspend the AMPure XP beads for use by vortexing about 30min in advance.
2. Add **appropriate volume** of resuspended AMPure XP beads to the reaction and mix by pipetting.
3. Incubate on a Hula mixer (rotator mixer) for 5 minutes at RT.
4. Prepare 5mL of fresh 70% ethanol in Nuclease-free water.
5. Spin down the sample and pellet on a magnet. Keep the tube on the magnet, and pipette off the supernatant.
6. Keep the tube on the magnet and wash the beads with 200 µL of freshly prepared 70% ethanol without disturbing the pellet. Remove the ethanol using a pipette and discard.
7. Repeat the previous step.
8. Spin down and place the tube back on the magnet. Pipette off any residual ethanol. Allow to dry for ~30 seconds, but do not dry the pellet to the point of cracking.
9. Remove the tube from the magnetic rack and resuspend pellet with **appropriate volume** 10 mM Tris-HCl (pH 8.0 with 50mM NaCl) or nuclease-free water. Incubate for 5 minutes at RT.
10. Pellet the beads on a magnet until the eluate is clear and colorless.
11. Remove and retain all of eluate into a clean 1.5 mL Eppendorf DNA LoBind tube.

Rapid adapter (RAP) ligation and loading library preparation (Universal)

1. Add 1 µL of RAP to the barcoded DNA.
2. Mix gently by flicking the tube, and spin down.
3. Incubate the reaction for 5 minutes at room temperature (RT).
4. Mix the following reagents in a 0.2 mL thin-walled PCR tube:

**Component**	**Volume**
Sequencing Buffer (SQB)	34 µL
Loading beads (LB)	25.5 µL
Nuclease-free water	4.5 µL
**Total**	**75 µL**

5. Priming and loading the SpotON flow cell.
