# Supplementary material for: Rapid Detection of Bacterial Pathogens and Antimicrobial Resistance Genes in Clinical Urine Samples With Urinary Tract Infection by Metagenomic Nanopore Sequencing
Source: Front Microbiol. 2022 May 17;13:858777. doi: 10.3389/fmicb.2022.858777 (PMC9152355; doi:10.3389/fmicb.2022.858777)
Supplement: Supplementary file 8 [file Image_1.pdf]

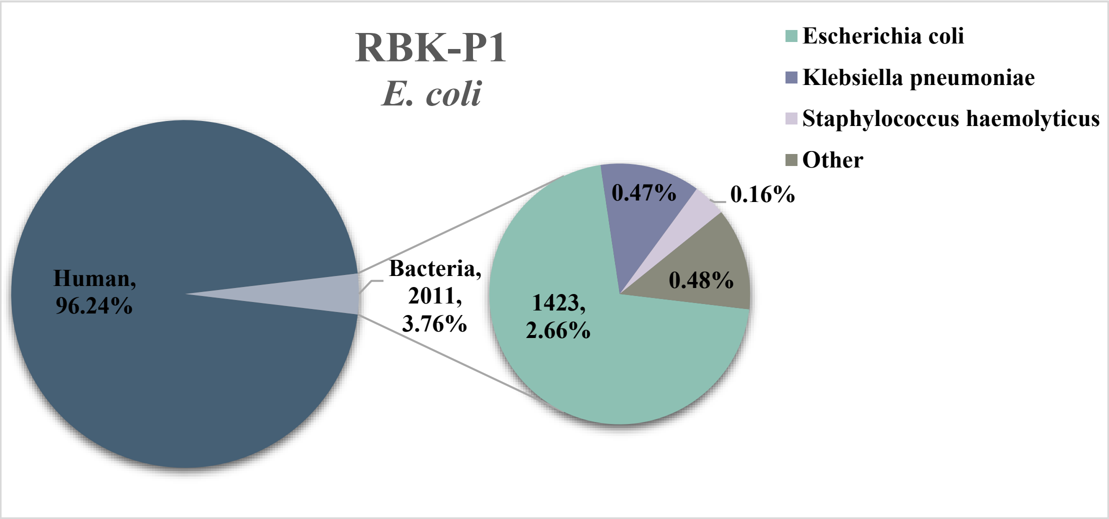
RBK-P1
E. coli
Escherichia coli
Klebsiella pneumoniae
Staphylococcus haemolyticus
Other
Human,
96.24%
Bacteria,
2011,
3.76%
1423,
2.66%
0.47%
0.16%
0.48%

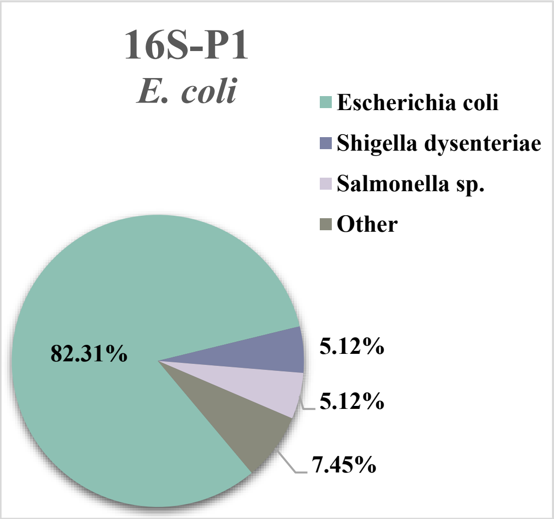
16S-P1
E. coli
Escherichia coli
Shigella dysenteriae
Salmonella sp.
Other
82.31%
5.12%
5.12%
7.45%

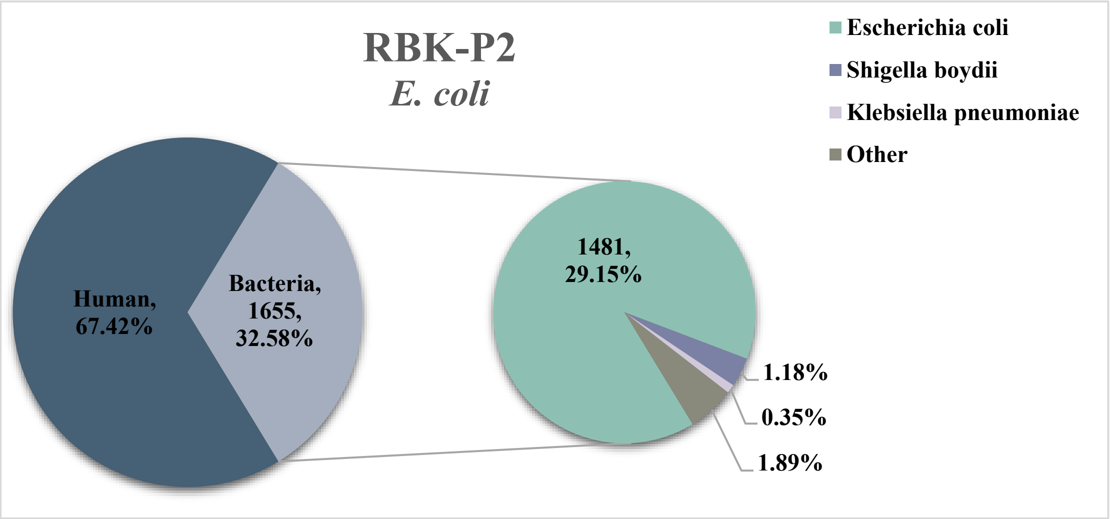
RBK-P2
E. coli
Escherichia coli
Shigella boydii
Klebsiella pneumoniae
Other
Human, 67.42%
Bacteria, 1655, 32.58%
1481, 29.15%
1.18%
0.35%
1.89%

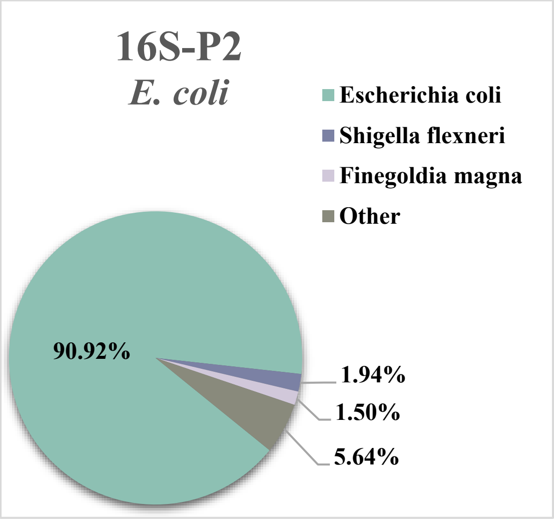
16S-P2
E. coli
Escherichia coli
Shigella flexneri
Finegoldia magna
Other
90.92%
1.94%
1.50%
5.64%

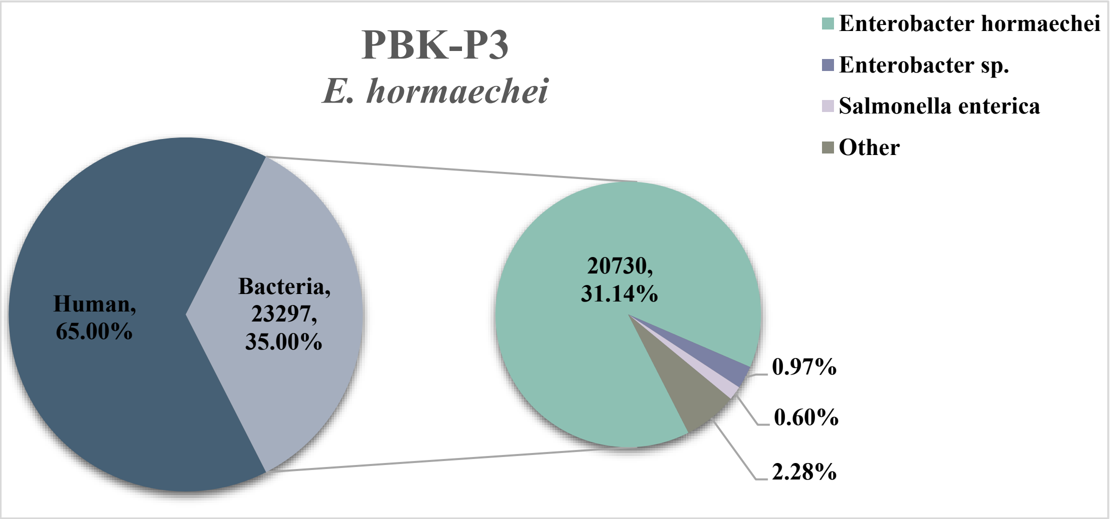

PBK-P3
E. hormaechei
Enterobacter hormaechei
Enterobacter sp.
Salmonella enterica
Other
Human, 65.00%
Bacteria, 23297, 35.00%
20730, 31.14%
0.97%
0.60%
2.28%

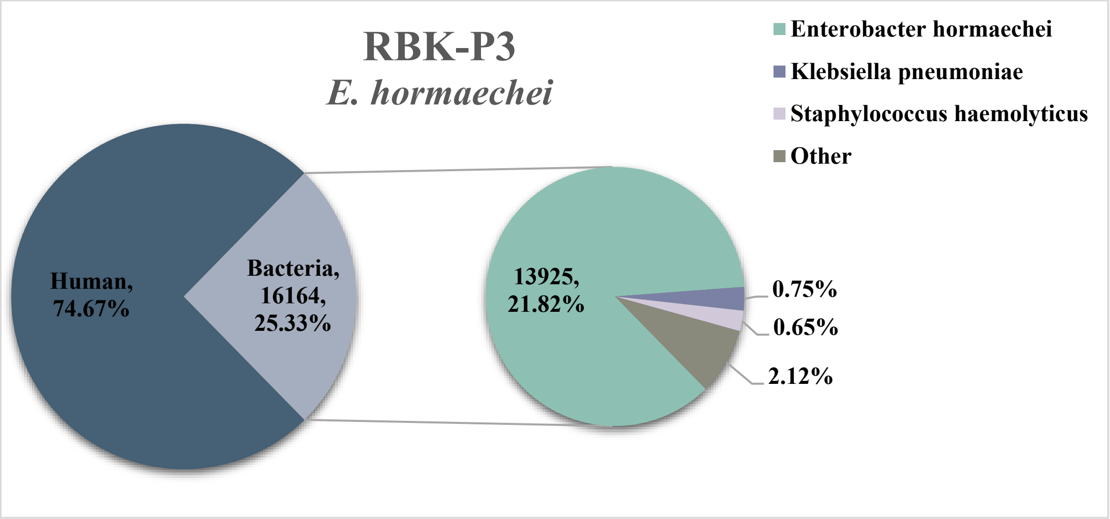

RBK-P3
E. hormaechei
Enterobacter hormaechei
Klebsiella pneumoniae
Staphylococcus haemolyticus
Other
Human, 74.67%
Bacteria, 16164, 25.33%
13925, 21.82%
0.75%
0.65%
2.12%

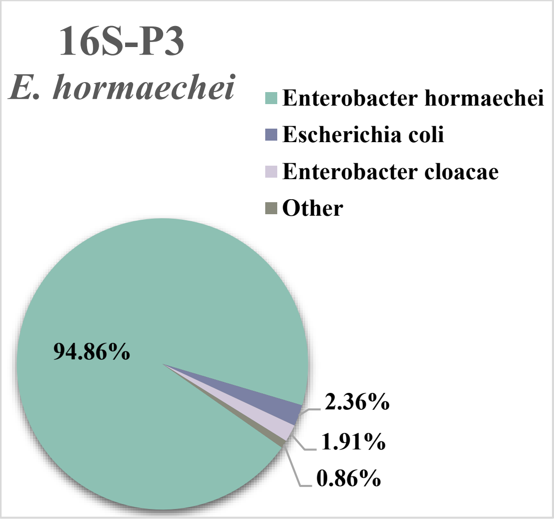

16S-P3
E. hormaechei
Enterobacter hormaechei
Escherichia coli
Enterobacter cloacae
Other
94.86%
2.36%
1.91%
0.86%

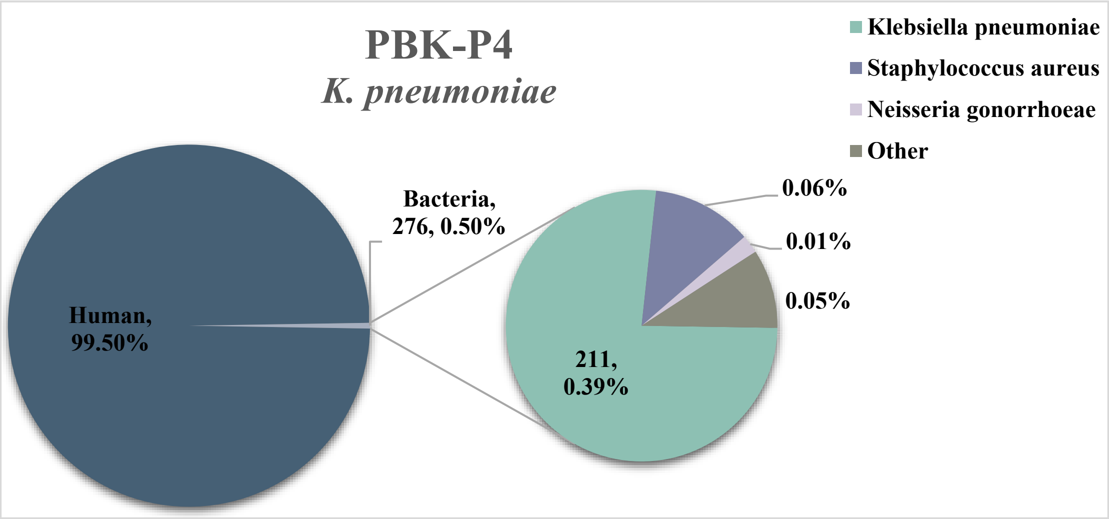
PBK-P4
K. pneumoniae
Klebsiella pneumoniae
Staphylococcus aureus
Neisseria gonorrhoeae
Other
Bacteria,
276, 0.50%
Human,
99.50%
0.06%
0.01%
0.05%
211,
0.39%

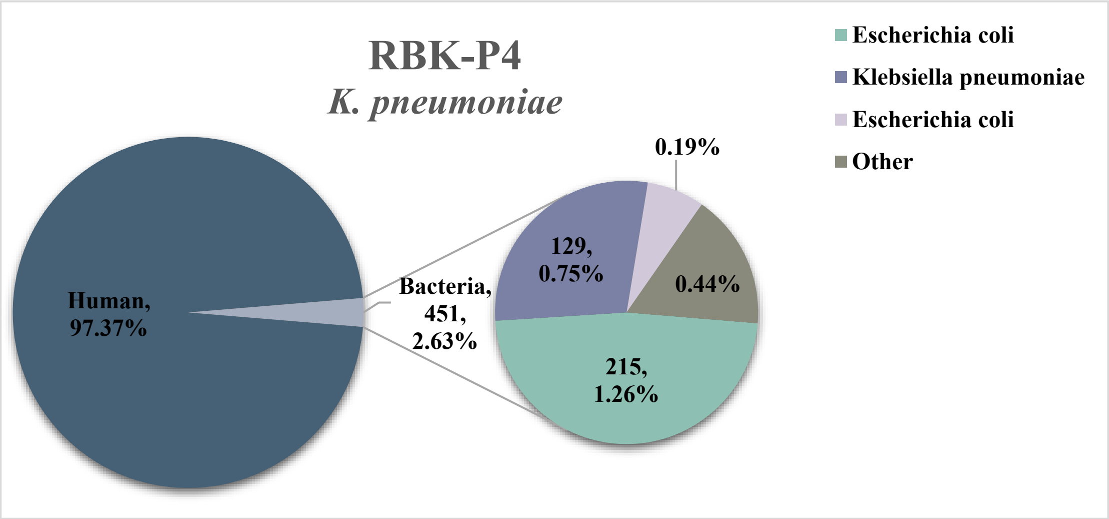
RBK-P4
K. pneumoniae
Escherichia coli
Klebsiella pneumoniae
Escherichia coli
Other
0.19%
Human,
97.37%
129,
0.75%
Bacteria,
451,
2.63%
0.44%
215,
1.26%

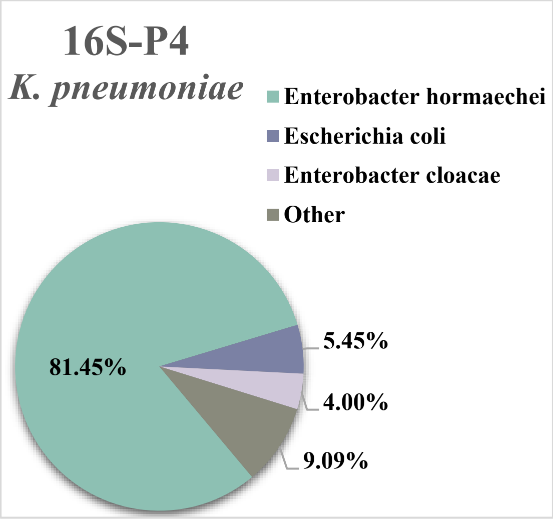
16S-P4
K. pneumoniae
Enterobacter hormaechei
Escherichia coli
Enterobacter cloacae
Other
81.45%
5.45%
4.00%
9.09%

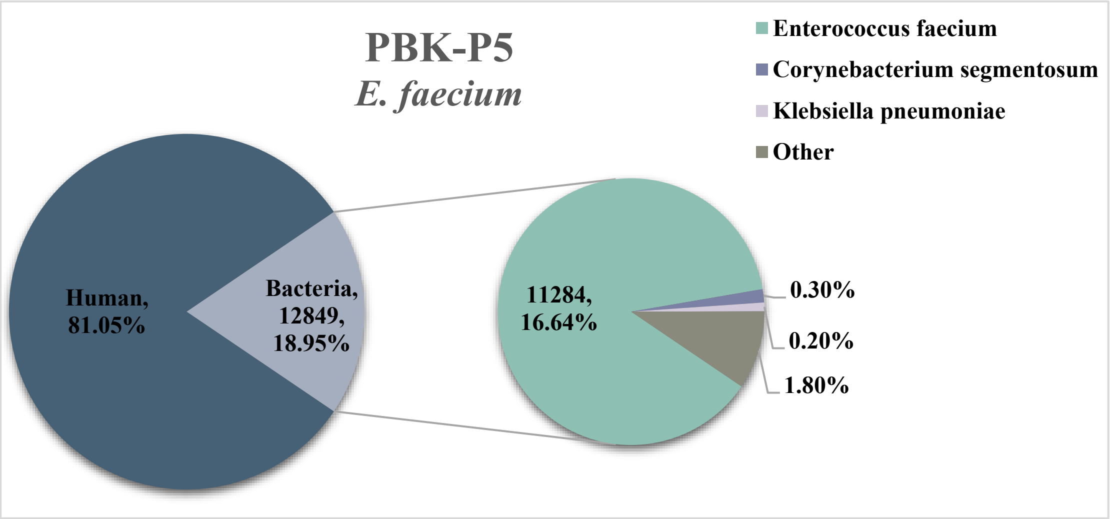
PBK-P5
E. faecium
Enterococcus faecium
Corynebacterium segmentosum
Klebsiella pneumoniae
Other
Human, 81.05%
Bacteria, 12849, 18.95%
11284, 16.64%
0.30%
0.20%
1.80%

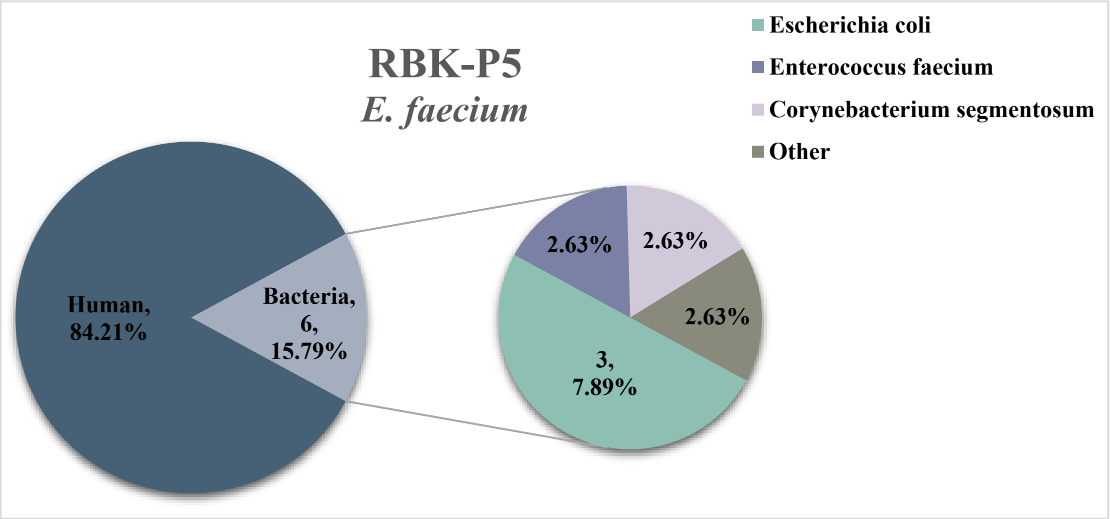
RBK-P5
E. faecium
Escherichia coli
Enterococcus faecium
Corynebacterium segmentosum
Other
Human, 84.21%
Bacteria, 6, 15.79%
2.63%
2.63%
2.63%
3, 7.89%

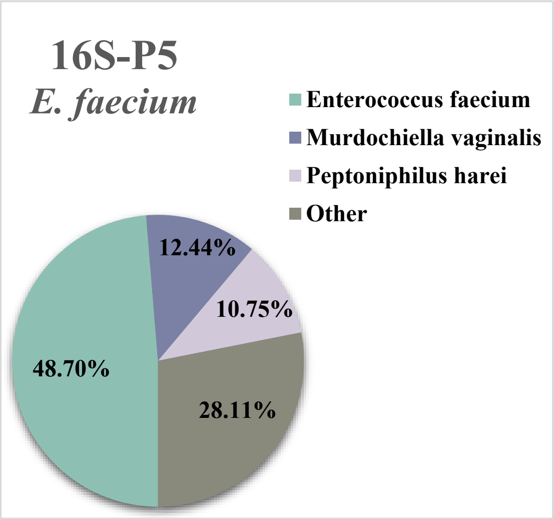
16S-P5
E. faecium
Enterococcus faecium
Murdochiella vaginalis
Peptoniphilus harei
Other
12.44%
10.75%
48.70%
28.11%

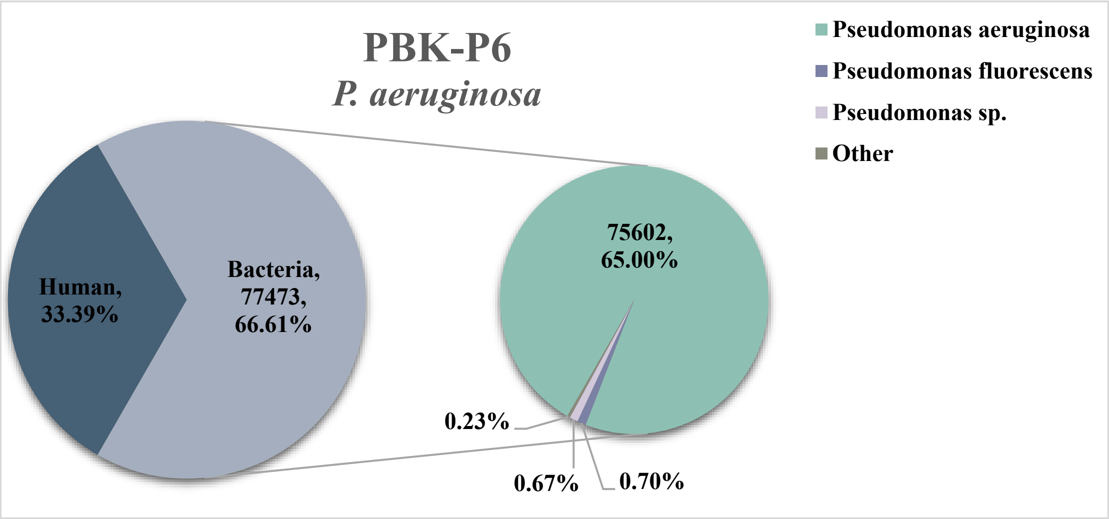
PBK-P6
P. aeruginosa
Pseudomonas aeruginosa
Pseudomonas fluorescens
Pseudomonas sp.
Other
Human, 33.39%
Bacteria, 77473, 66.61%
75602, 65.00%
0.23%
0.67%
0.70%

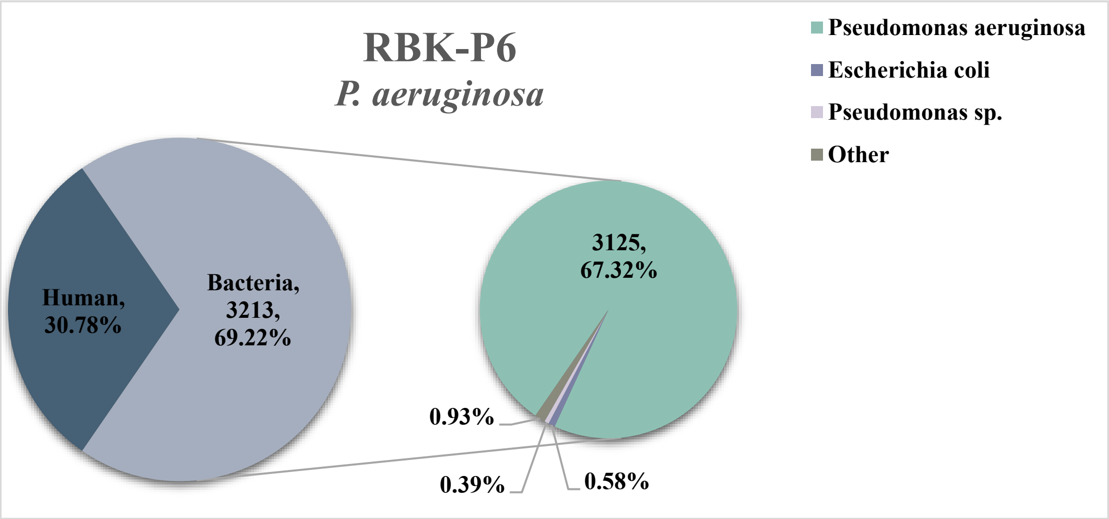
RBK-P6
P. aeruginosa
Pseudomonas aeruginosa
Escherichia coli
Pseudomonas sp.
Other
Human, 30.78%
Bacteria, 3213, 69.22%
3125, 67.32%
0.93%
0.39%
0.58%

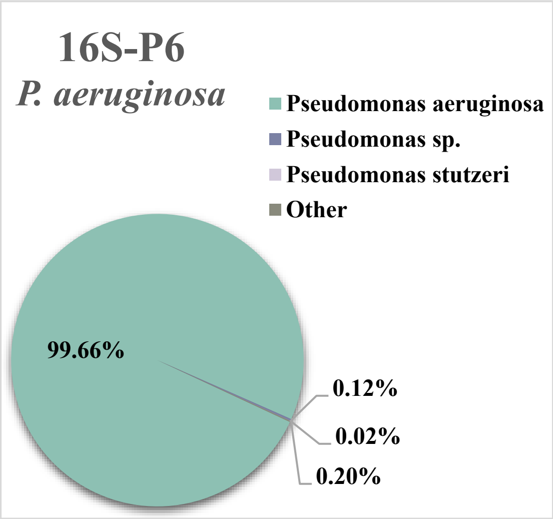
16S-P6
P. aeruginosa
Pseudomonas aeruginosa
Pseudomonas sp.
Pseudomonas stutzeri
Other
99.66%
0.12%
0.02%
0.20%

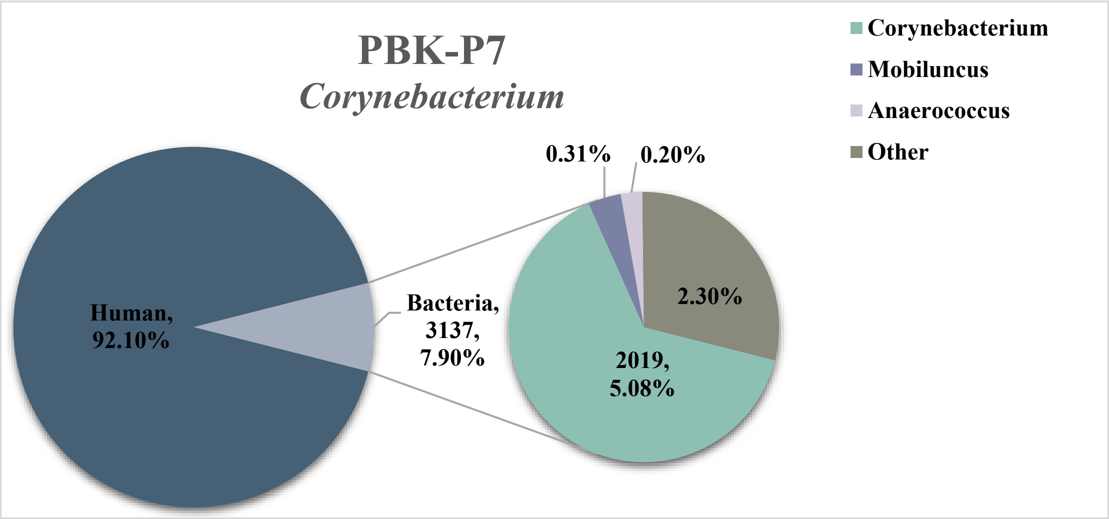
PBK-P7
Corynebacterium
Corynebacterium
Mobiluncus
Anaerococcus
Other
Human,
92.10%
Bacteria,
3137,
7.90%
0.31%
0.20%
2.30%
2019,
5.08%

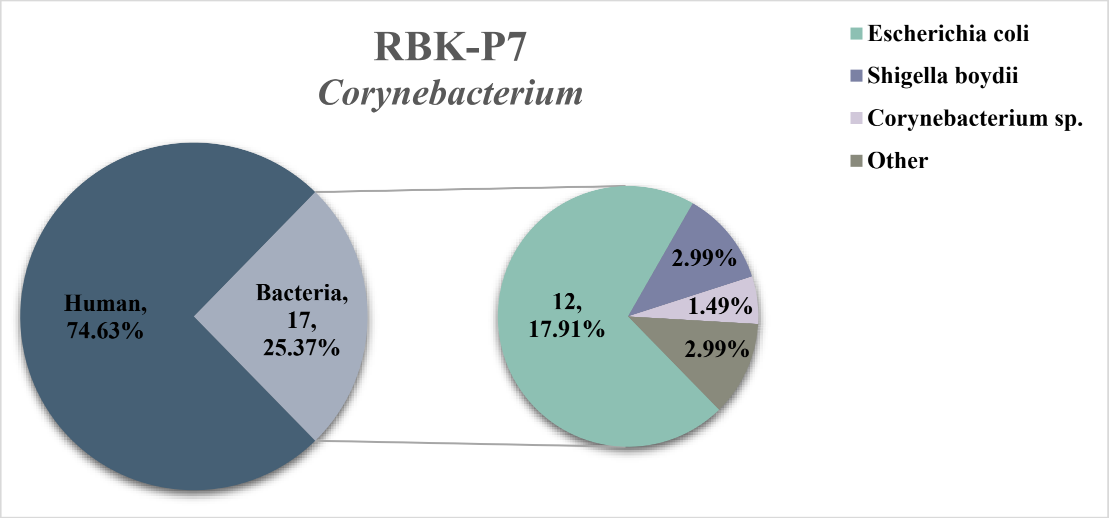
RBK-P7
Corynebacterium
Escherichia coli
Shigella boydii
Corynebacterium sp.
Other
Human,
74.63%
Bacteria,
17,
25.37%
12,
17.91%
2.99%
1.49%
2.99%

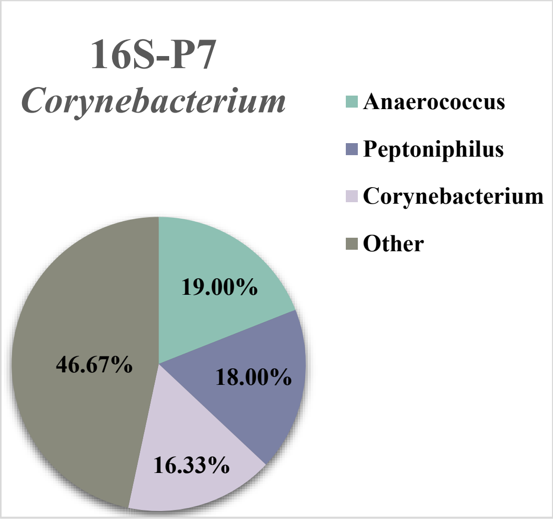
16S-P7
Corynebacterium
Anaerococcus
Peptoniphilus
Corynebacterium
Other
19.00%
18.00%
16.33%
46.67%

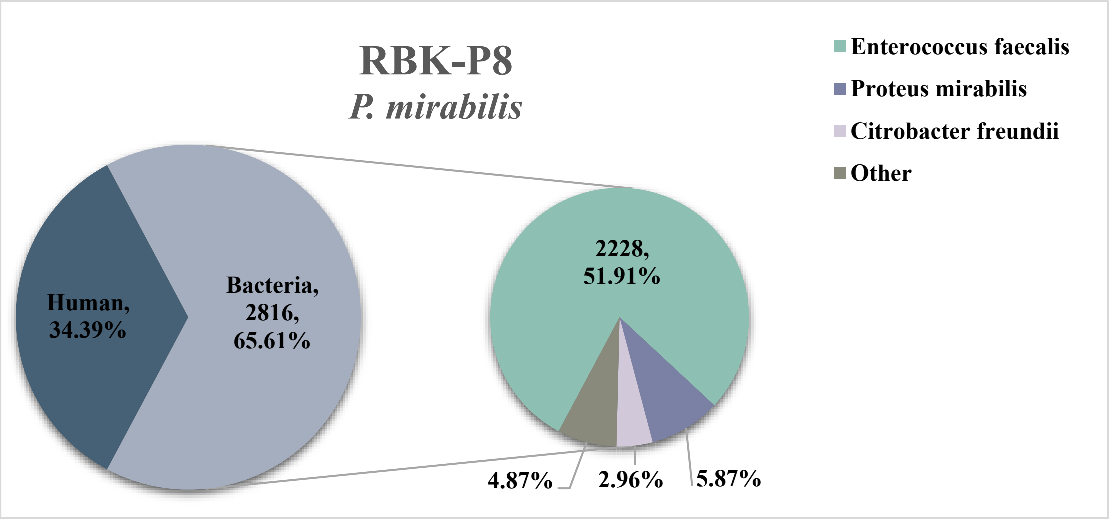
RBK-P8
P. mirabilis
Enterococcus faecalis
Proteus mirabilis
Citrobacter freundii
Other
Human,
34.39%
Bacteria,
2816,
65.61%
2228,
51.91%
4.87%
2.96%
5.87%

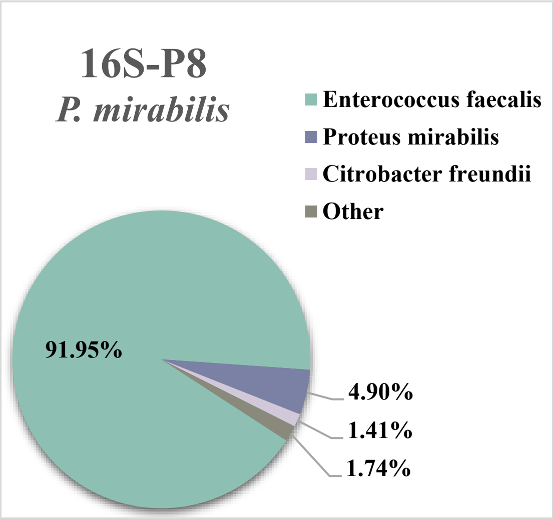
16S-P8
P. mirabilis
Enterococcus faecalis
Proteus mirabilis
Citrobacter freundii
Other
91.95%
4.90%
1.41%
1.74%

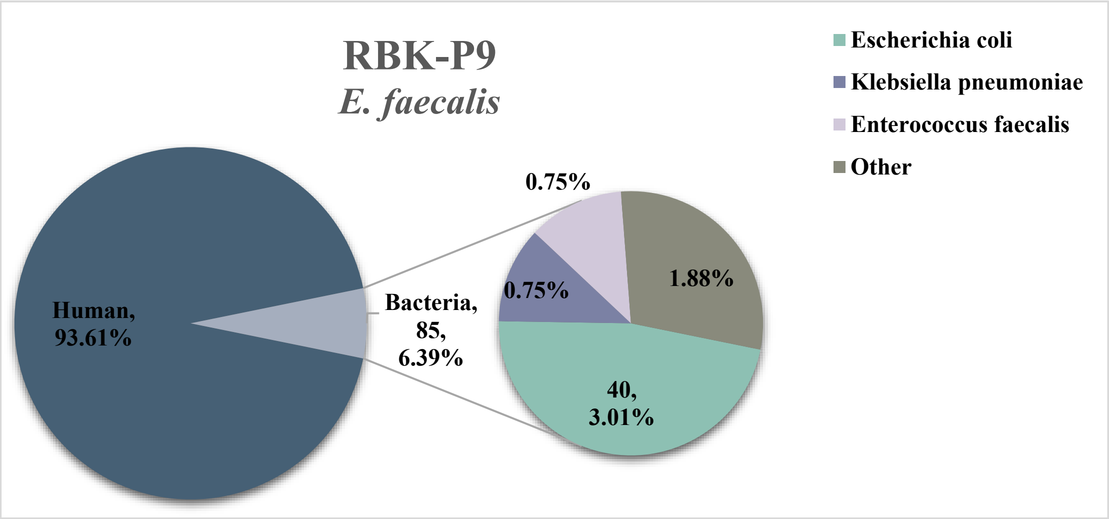
RBK-P9
E. faecalis
Escherichia coli
Klebsiella pneumoniae
Enterococcus faecalis
Other
Human,
93.61%
Bacteria,
85,
6.39%
0.75%
0.75%
1.88%
40,
3.01%

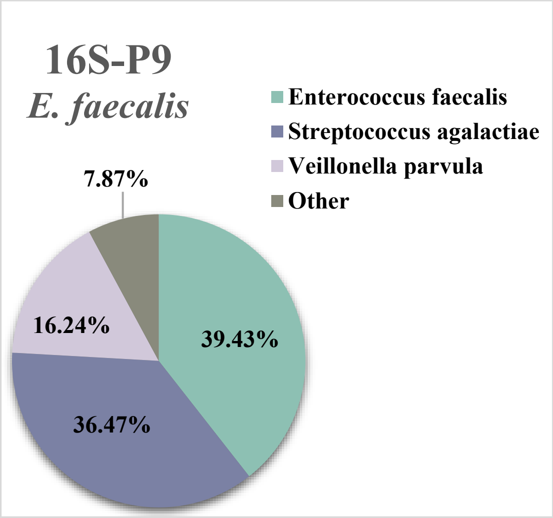
16S-P9
E. faecalis
Enterococcus faecalis
Streptococcus agalactiae
Veillonella parvula
Other
7.87%
16.24%
39.43%
36.47%

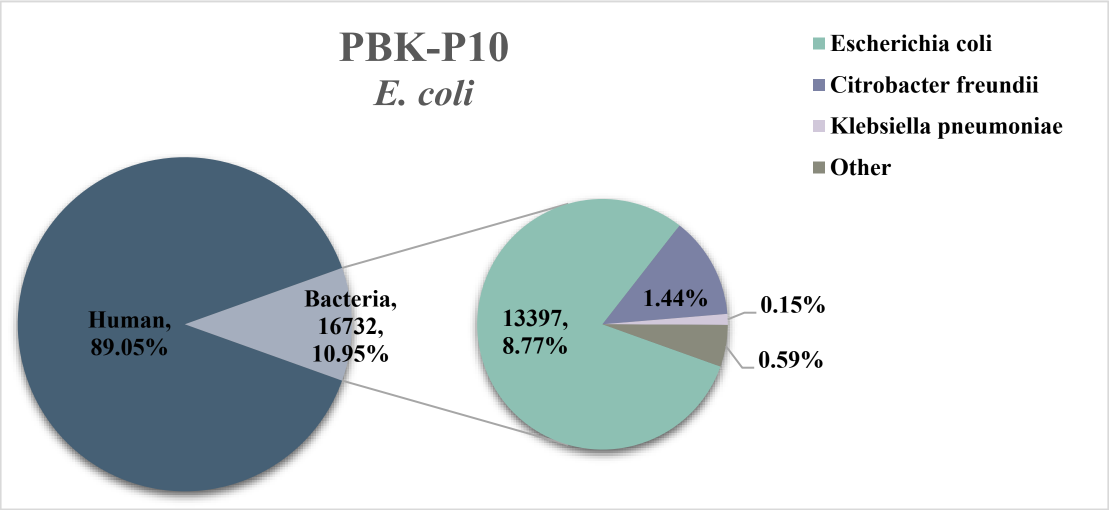
PBK-P10
E. coli
Escherichia coli
Citrobacter freundii
Klebsiella pneumoniae
Other
Human, 89.05%
Bacteria, 16732, 10.95%
13397, 8.77%
1.44%
0.15%
0.59%

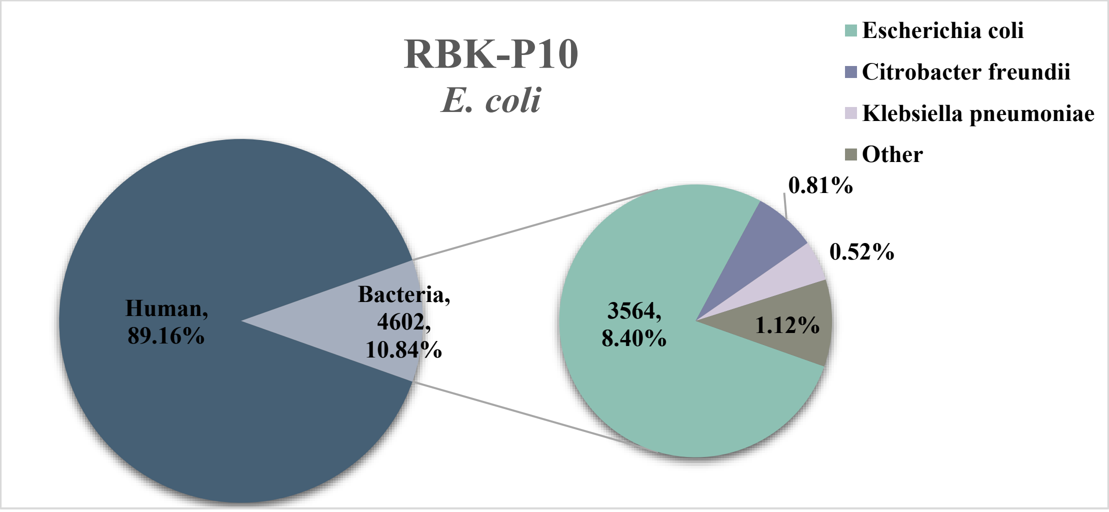
RBK-P10
E. coli
Escherichia coli
Citrobacter freundii
Klebsiella pneumoniae
Other
Human, 89.16%
Bacteria, 4602, 10.84%
3564, 8.40%
0.81%
0.52%
1.12%

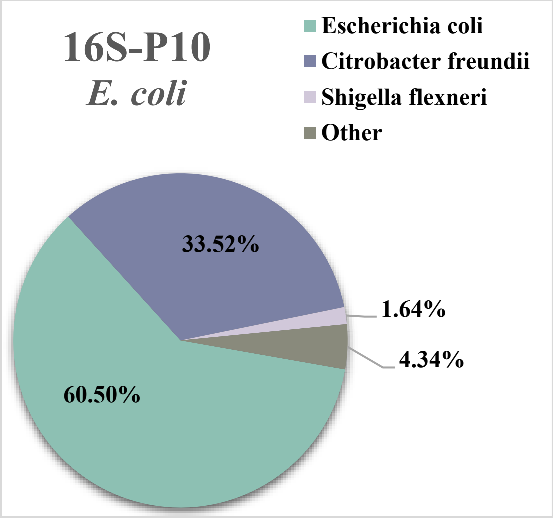
16S-P10
E. coli
Escherichia coli
Citrobacter freundii
Shigella flexneri
Other
33.52%
1.64%
4.34%
60.50%

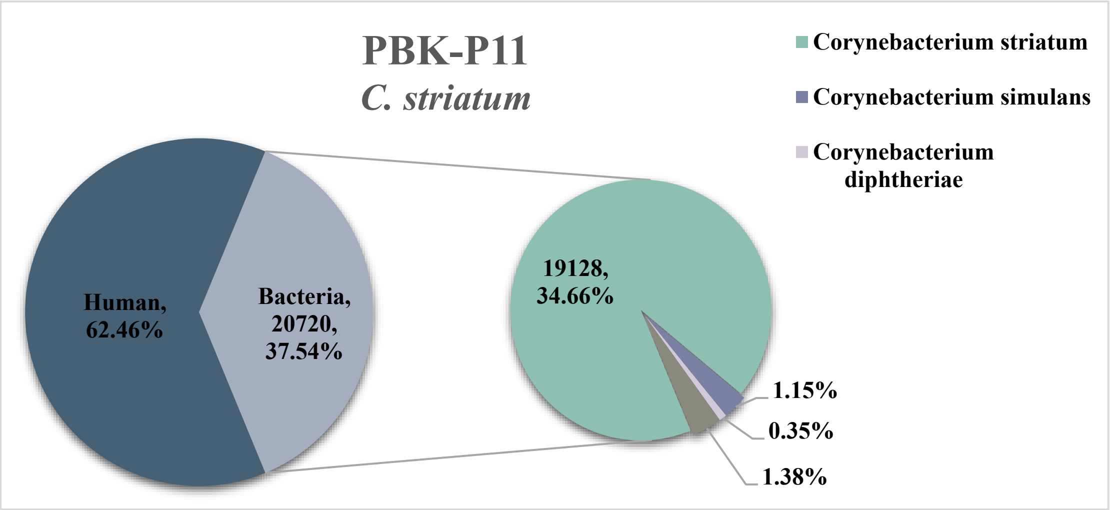
PBK-P11
C. striatum
Corynebacterium striatum
Corynebacterium simulans
Corynebacterium diphtheriae
Human, 62.46%
Bacteria, 20720, 37.54%
19128, 34.66%
1.15%
0.35%
1.38%

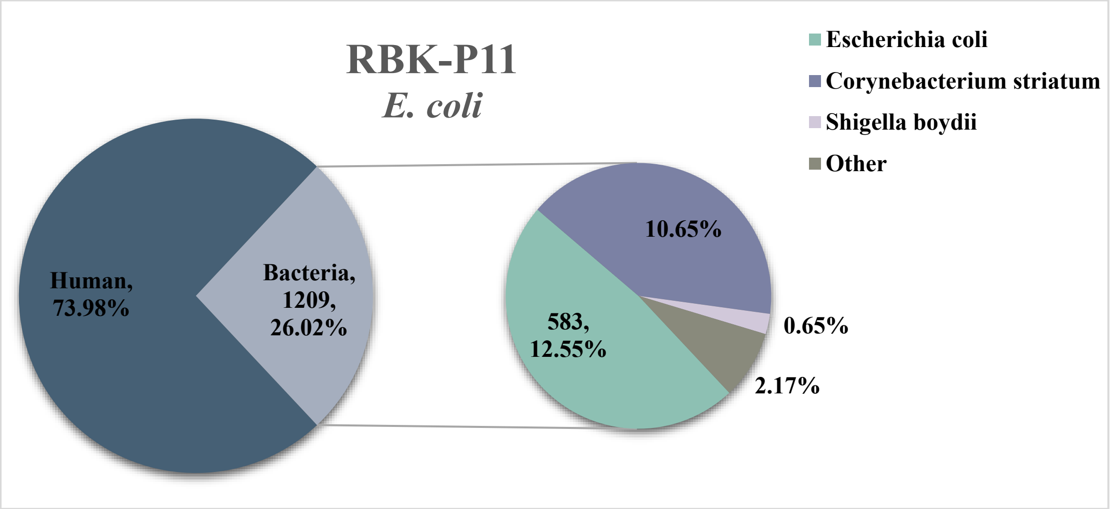
RBK-P11
E. coli
Escherichia coli
Corynebacterium striatum
Shigella boydii
Other
Human, 73.98%
Bacteria, 1209, 26.02%
10.65%
583, 12.55%
0.65%
2.17%

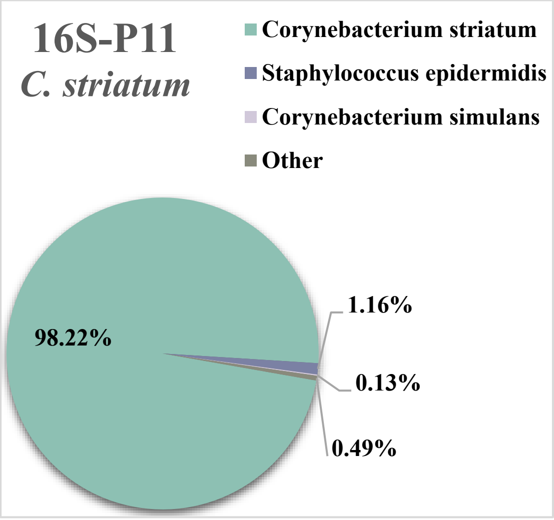
16S-P11
C. striatum
Corynebacterium striatum
Staphylococcus epidermidis
Corynebacterium simulans
Other
98.22%
1.16%
0.13%
0.49%

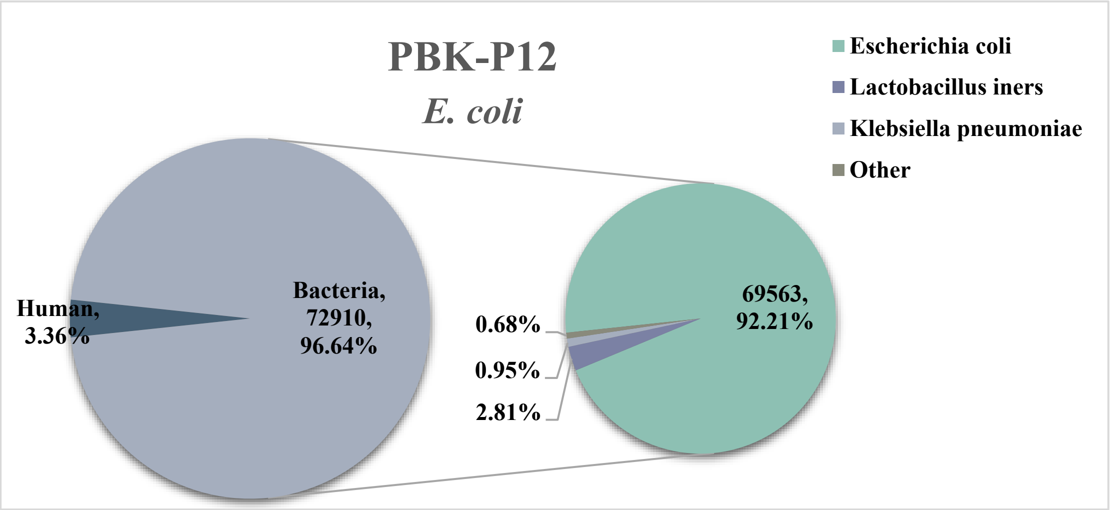
PBK-P12
E. coli
Escherichia coli
Lactobacillus iners
Klebsiella pneumoniae
Other
Human,
3.36%
Bacteria,
72910,
96.64%
69563,
92.21%
0.68%
0.95%
2.81%

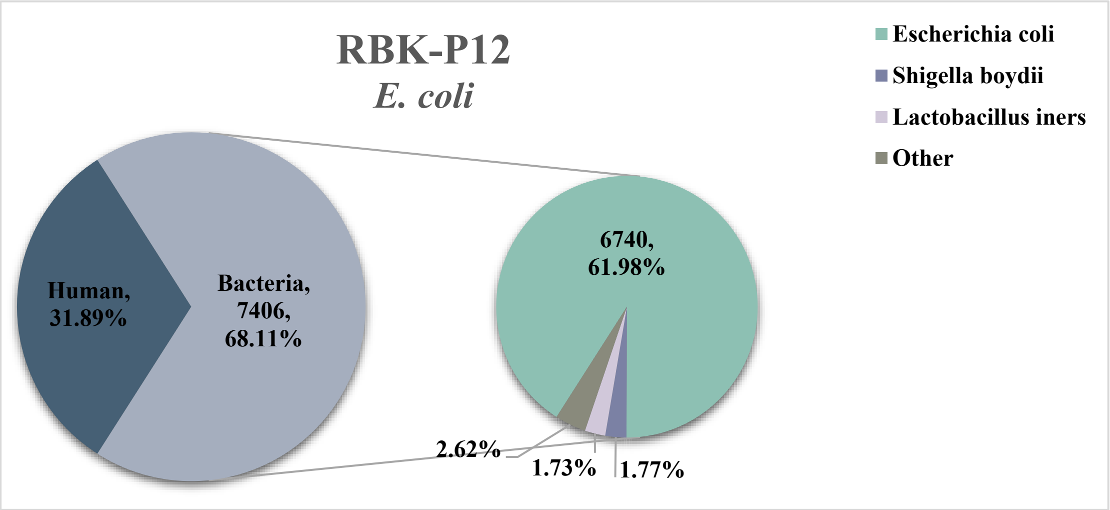
RBK-P12
E. coli
Escherichia coli
Shigella boydii
Lactobacillus iners
Other
Human,
31.89%
Bacteria,
7406,
68.11%
6740,
61.98%
2.62%
1.73%
1.77%

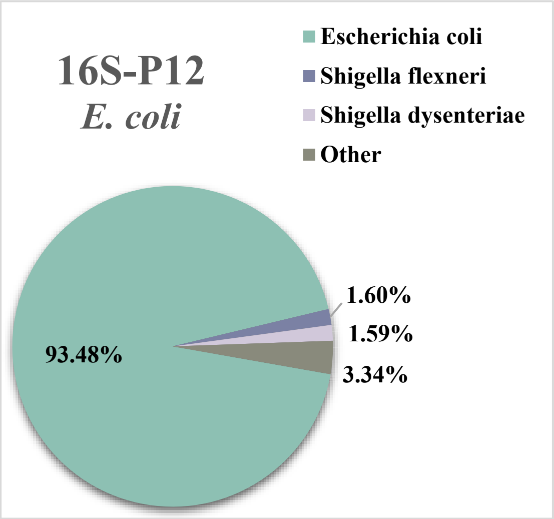
16S-P12
E. coli
Escherichia coli
Shigella flexneri
Shigella dysenteriae
Other
93.48%
1.60%
1.59%
3.34%

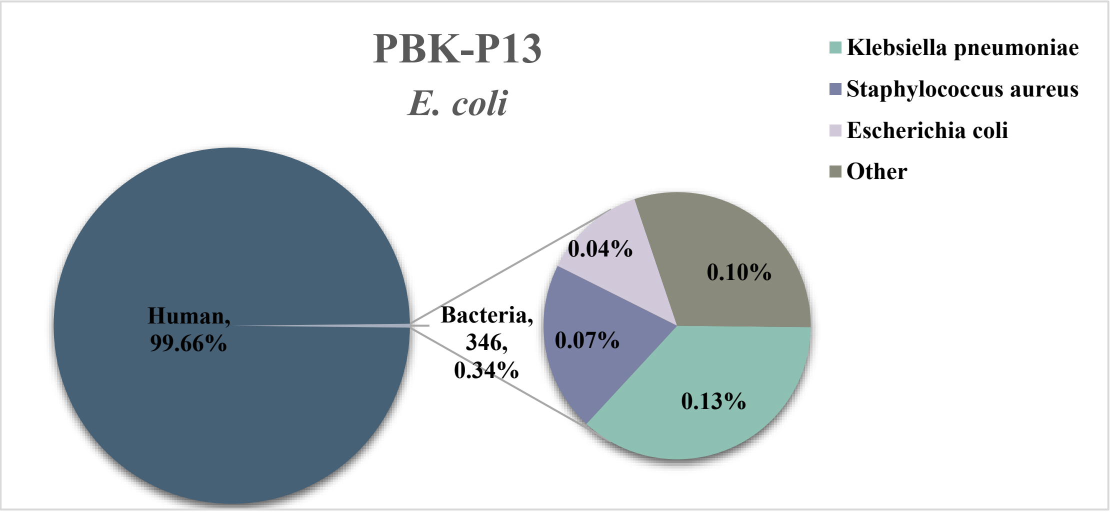
PBK-P13
E. coli
Klebsiella pneumoniae
Staphylococcus aureus
Escherichia coli
Other
Human,
99.66%
Bacteria,
346,
0.34%
0.04%
0.10%
0.07%
0.13%

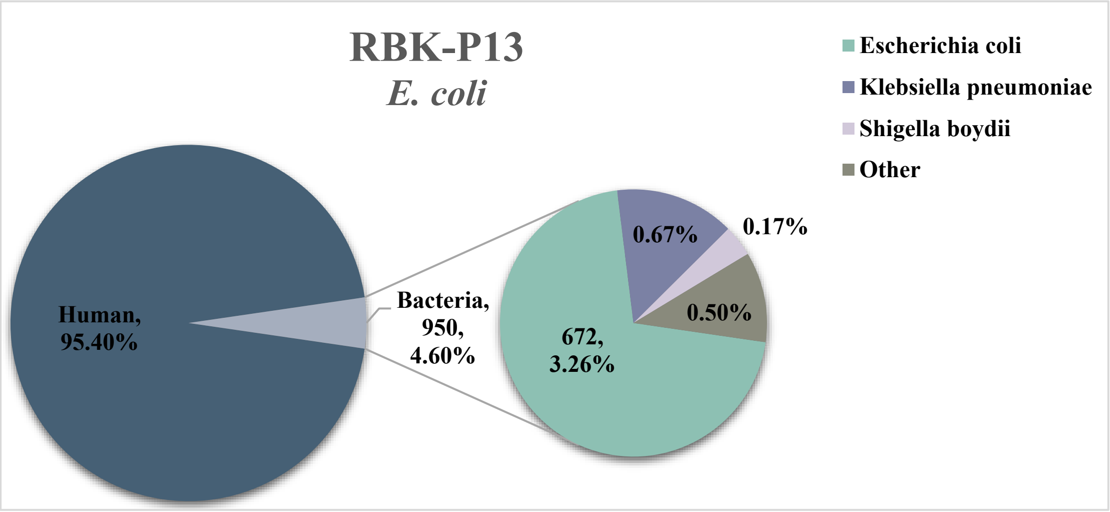
RBK-P13
E. coli
Escherichia coli
Klebsiella pneumoniae
Shigella boydii
Other
Human,
95.40%
Bacteria,
950,
4.60%
0.67%
0.17%
0.50%
672,
3.26%

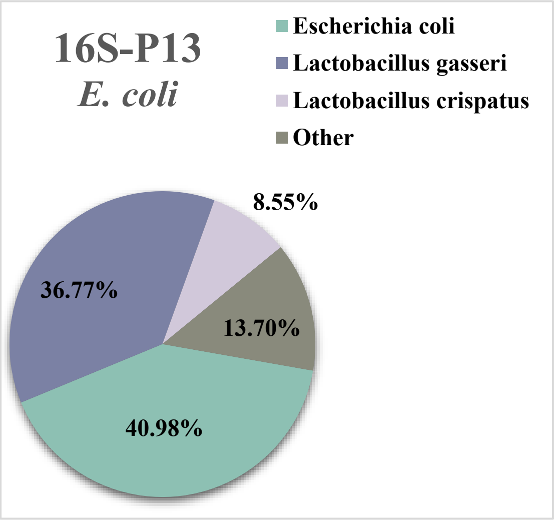
16S-P13
E. coli
Escherichia coli
Lactobacillus gasseri
Lactobacillus crispatus
Other
8.55%
36.77%
13.70%
40.98%

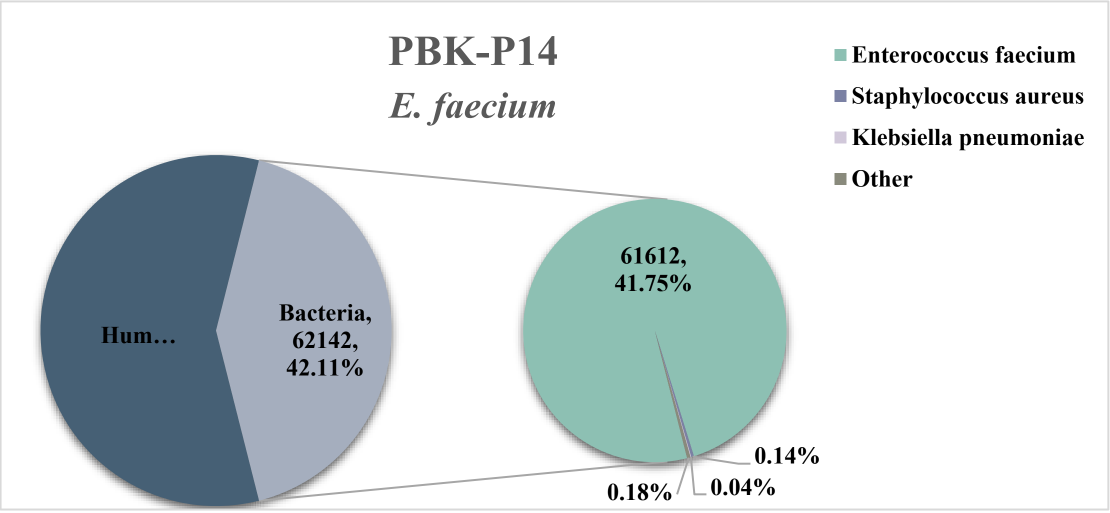
PBK-P14
E. faecium
Enterococcus faecium
Staphylococcus aureus
Klebsiella pneumoniae
Other
Hum…
Bacteria, 62142, 42.11%
61612, 41.75%
0.14%
0.18%
0.04%

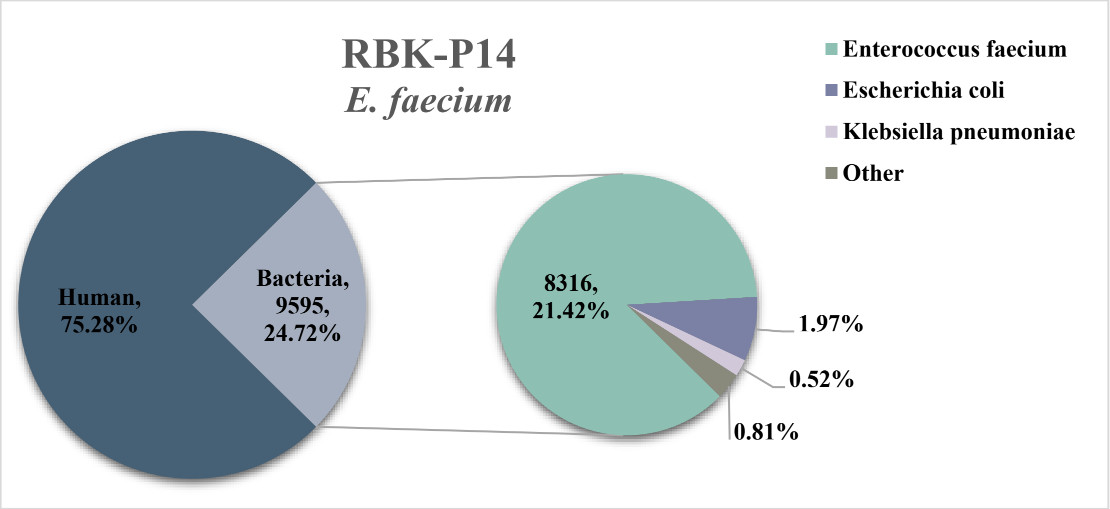
RBK-P14
E. faecium
Enterococcus faecium
Escherichia coli
Klebsiella pneumoniae
Other
Human, 75.28%
Bacteria, 9595, 24.72%
8316, 21.42%
1.97%
0.52%
0.81%

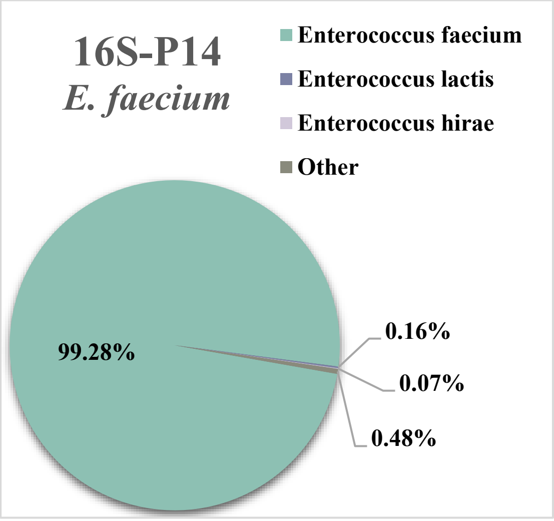
16S-P14
E. faecium
Enterococcus faecium
Enterococcus lactis
Enterococcus hirae
Other
99.28%
0.16%
0.07%
0.48%

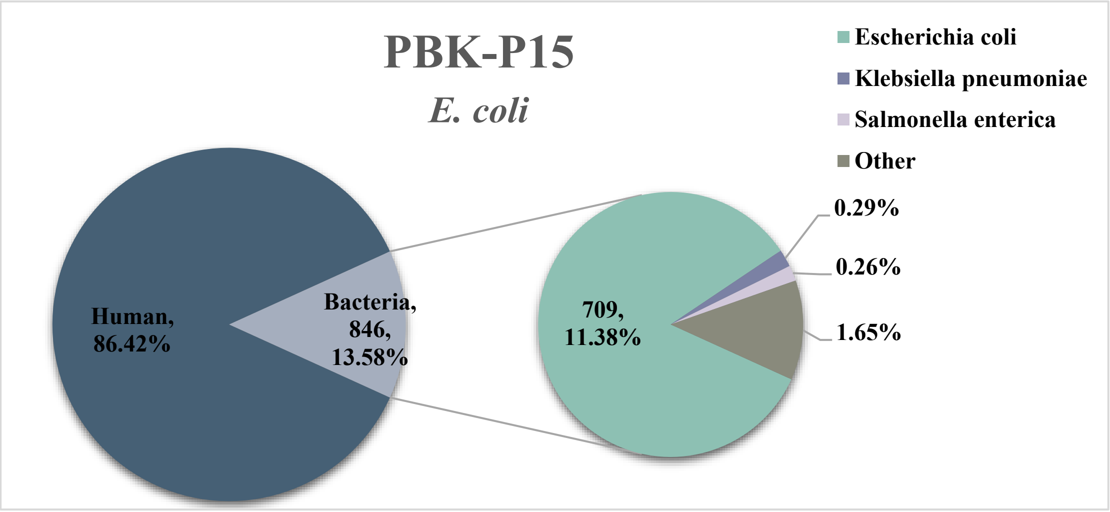
PBK-P15
E. coli
Escherichia coli
Klebsiella pneumoniae
Salmonella enterica
Other
Human, 86.42%
Bacteria, 846, 13.58%
709, 11.38%
0.29%
0.26%
1.65%

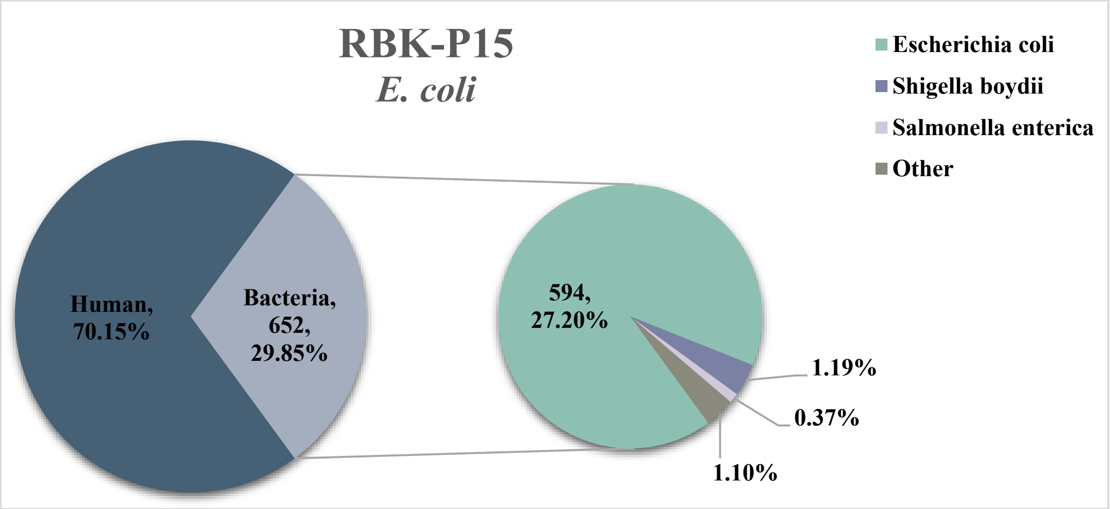
RBK-P15
E. coli
Escherichia coli
Shigella boydii
Salmonella enterica
Other
Human, 70.15%
Bacteria, 652, 29.85%
594, 27.20%
1.19%
0.37%
1.10%

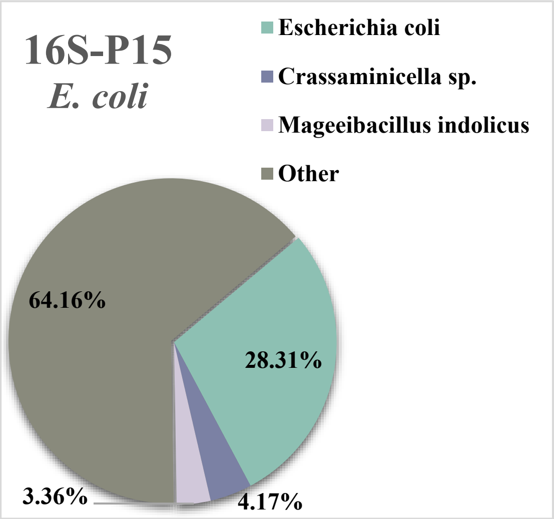
16S-P15
E. coli
Escherichia coli
Crassaminicella sp.
Mageeibacillus indolicus
Other
64.16%
28.31%
3.36%
4.17%

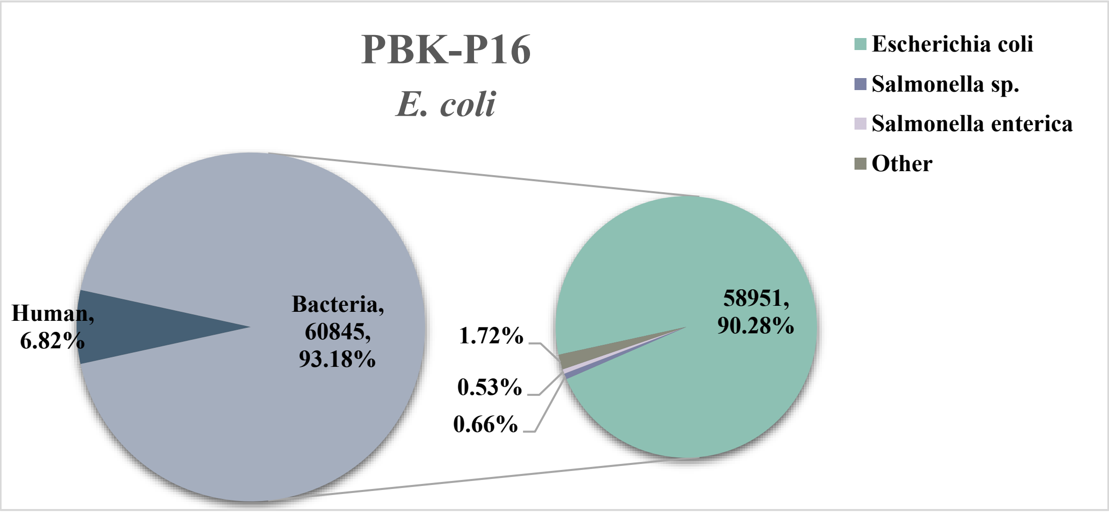
PBK-P16
E. coli
Escherichia coli
Salmonella sp.
Salmonella enterica
Other
Human,
6.82%
Bacteria,
60845,
93.18%
58951,
90.28%
1.72%
0.53%
0.66%

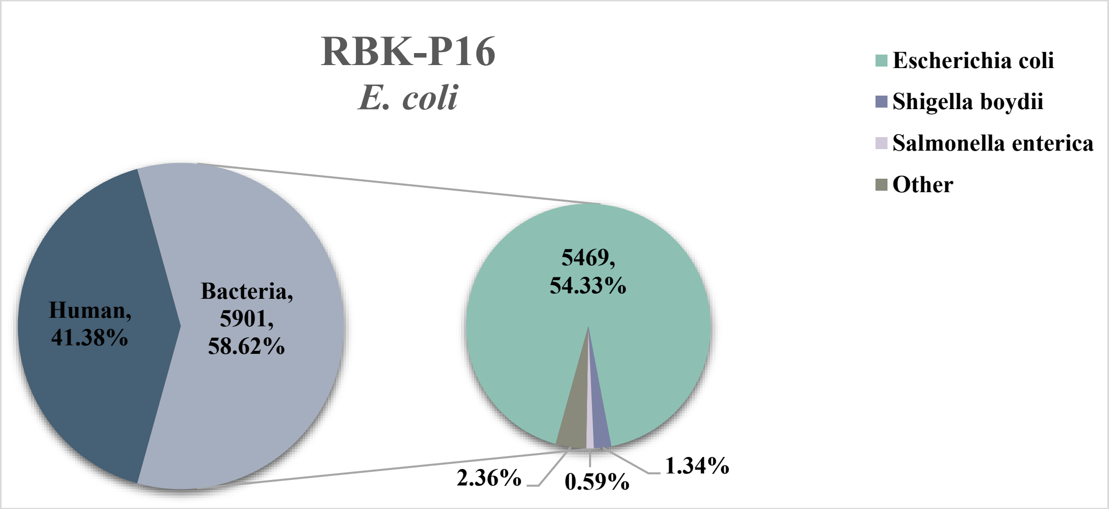
RBK-P16
E. coli
Escherichia coli
Shigella boydii
Salmonella enterica
Other
Human,
41.38%
Bacteria,
5901,
58.62%
5469,
54.33%
2.36%
0.59%
1.34%

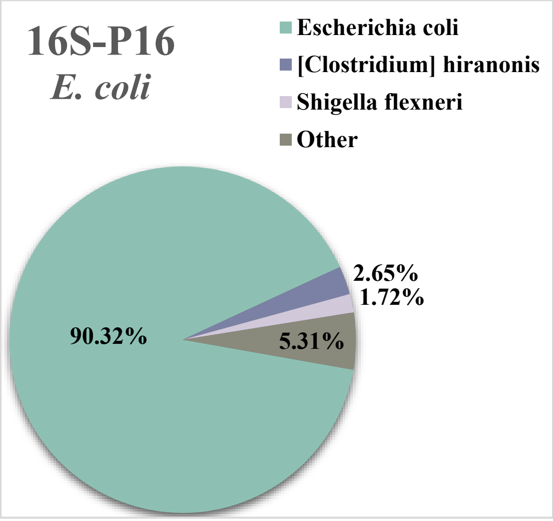
16S-P16
E. coli
Escherichia coli
[Clostridium] hiranonis
Shigella flexneri
Other
90.32%
2.65%
1.72%
5.31%

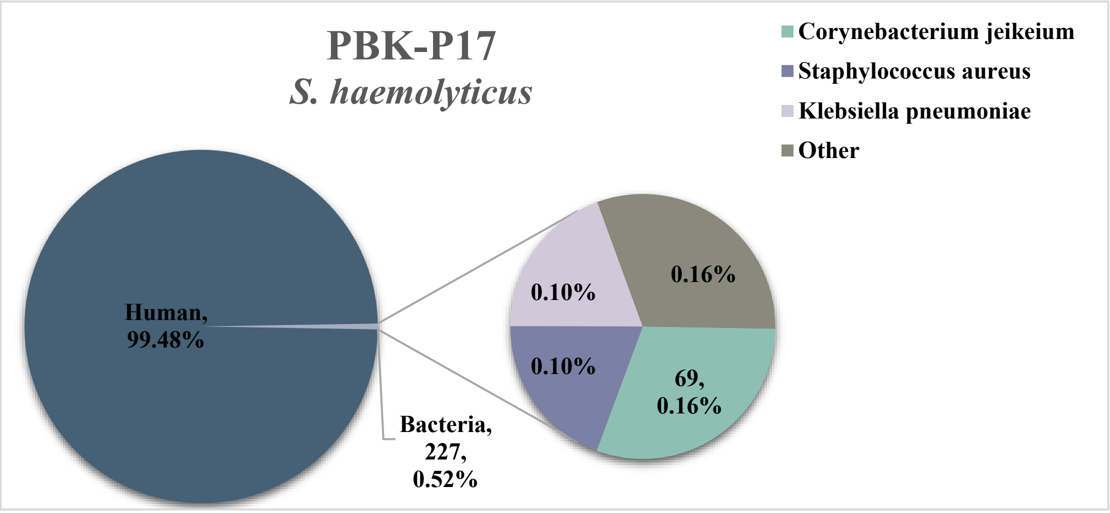
PBK-P17
S. haemolyticus
Corynebacterium jeikeium
Staphylococcus aureus
Klebsiella pneumoniae
Other
Human, 99.48%
Bacteria, 227, 0.52%
0.10%
0.16%
0.10%
69, 0.16%

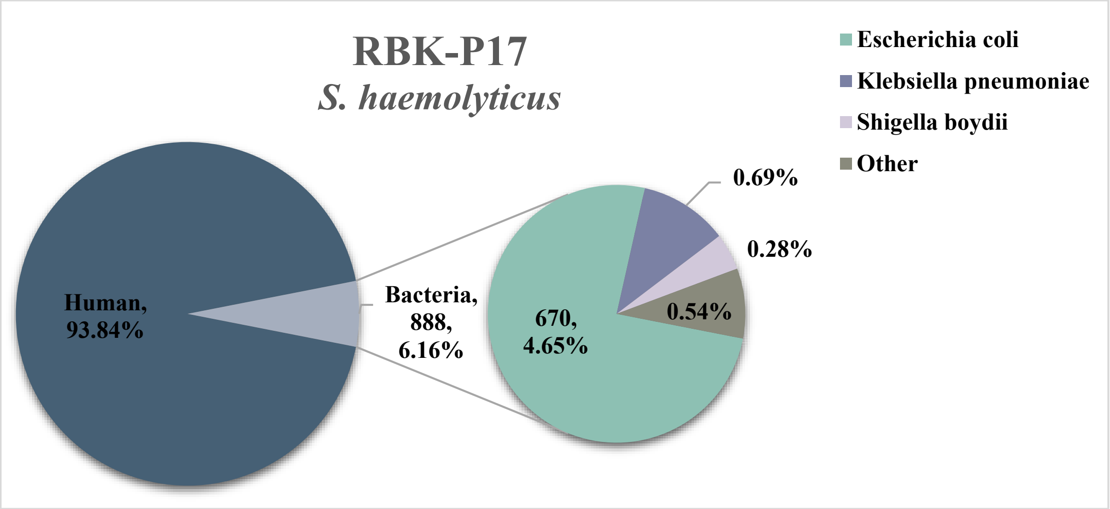
RBK-P17
S. haemolyticus
Escherichia coli
Klebsiella pneumoniae
Shigella boydii
Other
Human, 93.84%
Bacteria, 888, 6.16%
0.69%
0.28%
0.54%
670, 4.65%

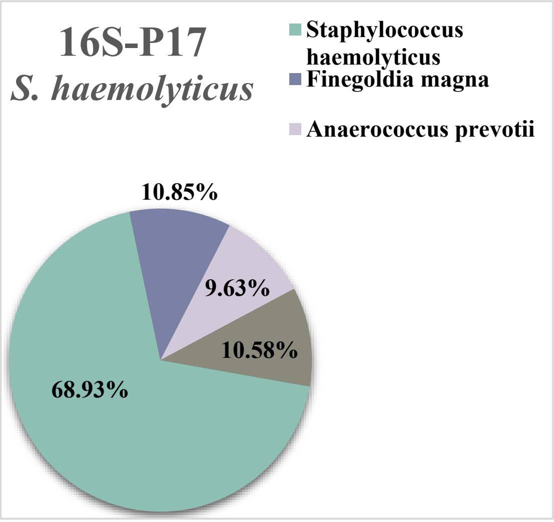
16S-P17
S. haemolyticus
Staphylococcus haemolyticus
Finegoldia magna
Anaerococcus prevotii
10.85%
9.63%
10.58%
68.93%

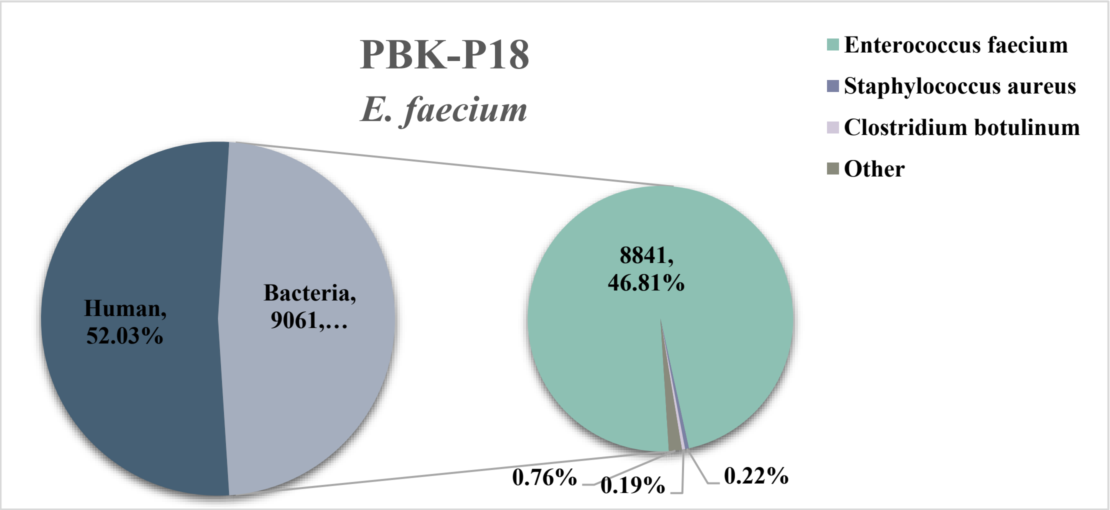
PBK-P18
E. faecium
Enterococcus faecium
Staphylococcus aureus
Clostridium botulinum
Other
Human, 52.03%
Bacteria, 9061,...
8841, 46.81%
0.76%
0.19%
0.22%

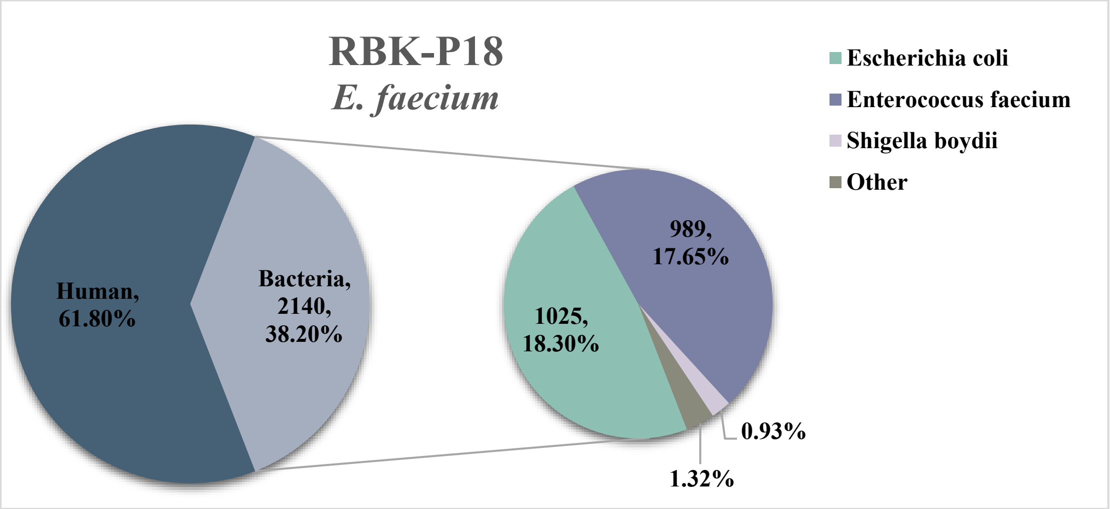
RBK-P18
E. faecium
Escherichia coli
Enterococcus faecium
Shigella boydii
Other
Human, 61.80%
Bacteria, 2140, 38.20%
989, 17.65%
1025, 18.30%
0.93%
1.32%

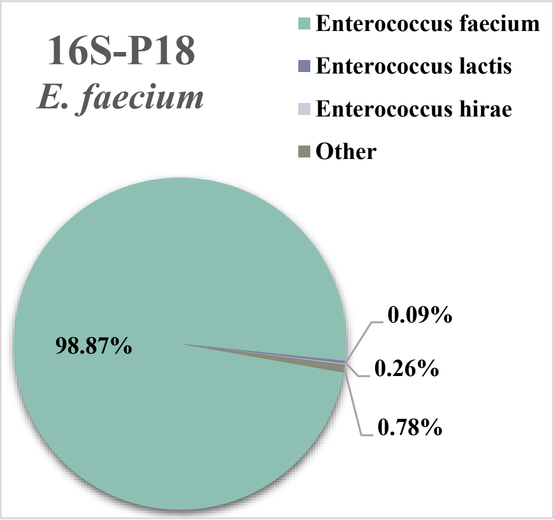
16S-P18
E. faecium
Enterococcus faecium
Enterococcus lactis
Enterococcus hirae
Other
98.87%
0.09%
0.26%
0.78%

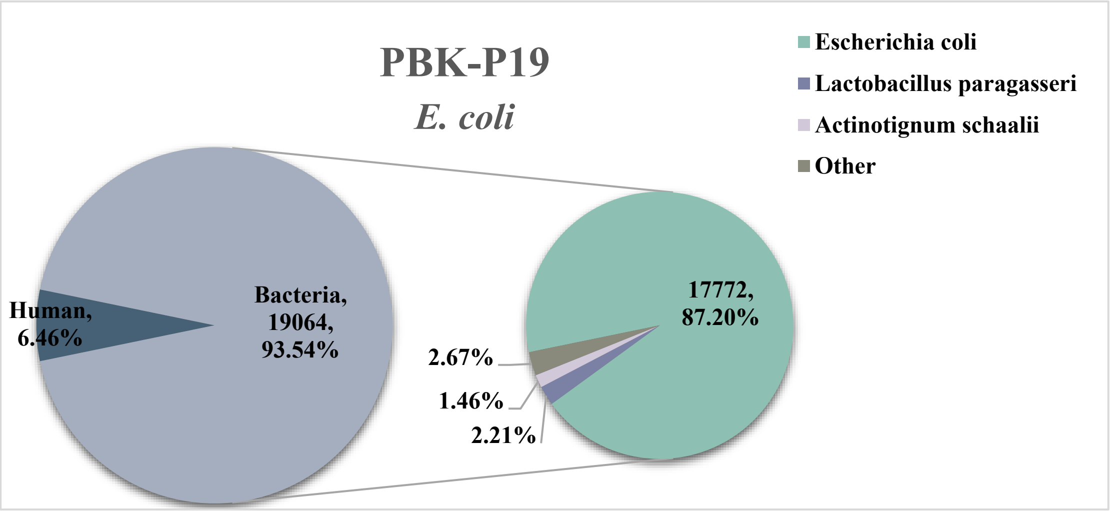
PBK-P19
E. coli
Escherichia coli
Lactobacillus paragasseri
Actinotignum schaalii
Other
Human, 6.46%
Bacteria, 19064, 93.54%
17772, 87.20%
2.67%
1.46%
2.21%

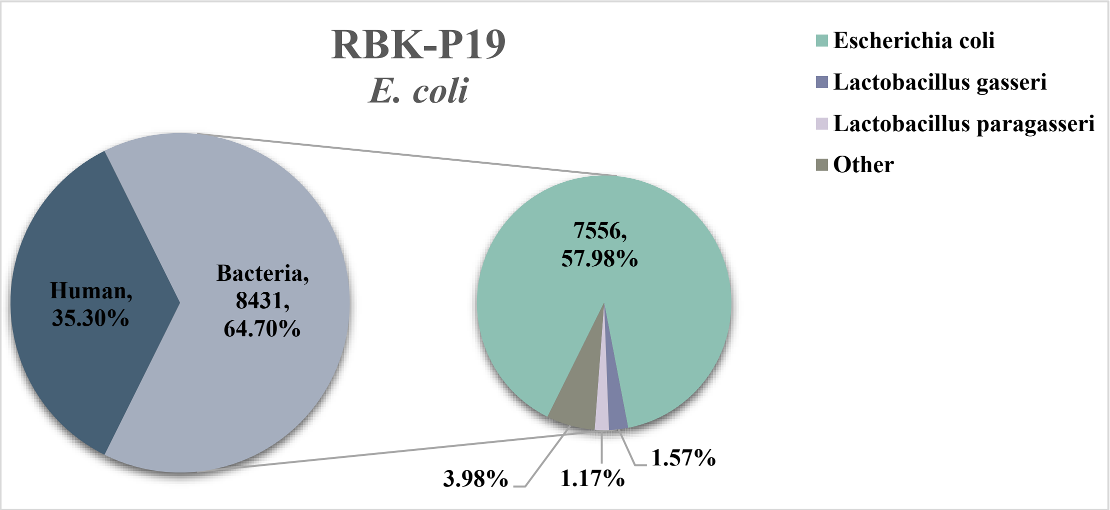
RBK-P19
E. coli
Escherichia coli
Lactobacillus gasseri
Lactobacillus paragasseri
Other
Human, 35.30%
Bacteria, 8431, 64.70%
7556, 57.98%
3.98%
1.17%
1.57%

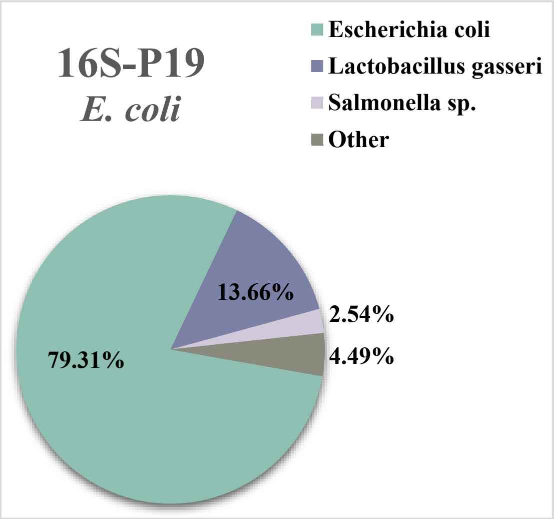
16S-P19
E. coli
Escherichia coli
Lactobacillus gasseri
Salmonella sp.
Other
13.66%
2.54%
4.49%
79.31%

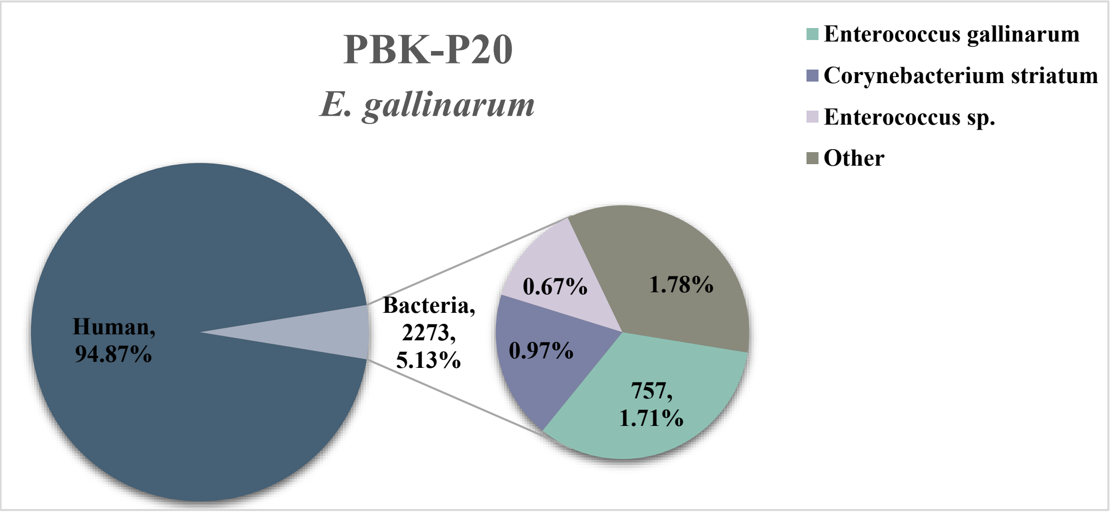
PBK-P20
E. gallinarum
Enterococcus gallinarum
Corynebacterium striatum
Enterococcus sp.
Other
Human, 94.87%
Bacteria, 2273, 5.13%
0.67%
1.78%
0.97%
757, 1.71%

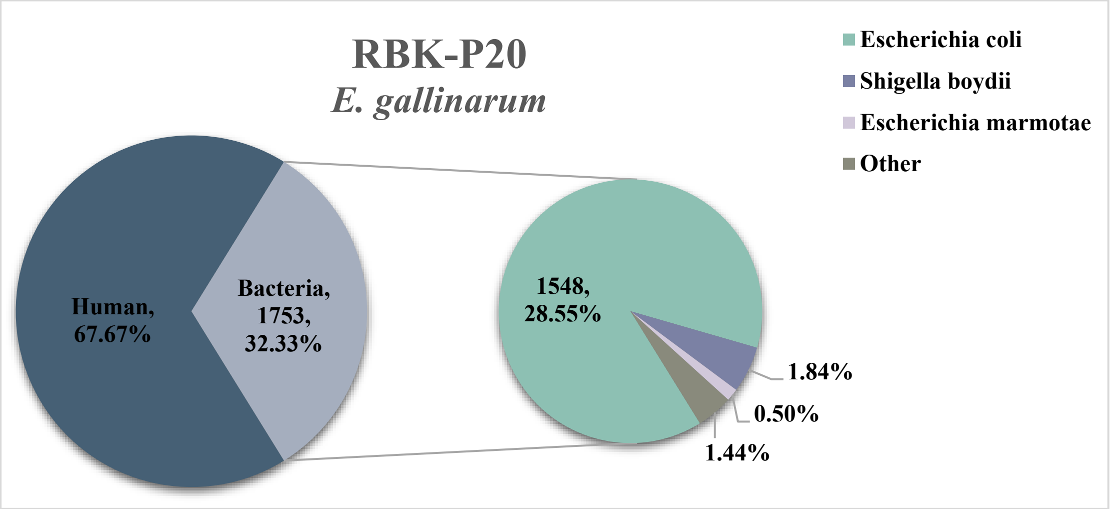
RBK-P20
E. gallinarum
Escherichia coli
Shigella boydii
Escherichia marmotae
Other
Human, 67.67%
Bacteria, 1753, 32.33%
1548, 28.55%
1.84%
0.50%
1.44%

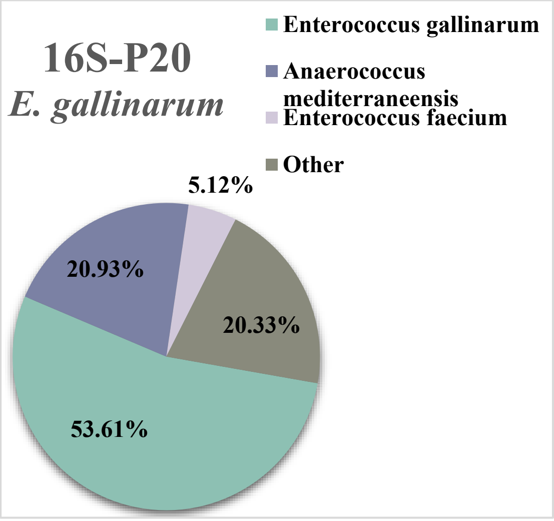
16S-P20
E. gallinarum
Enterococcus gallinarum
Anaerococcus mediterraneensis
Enterococcus faecium
Other
5.12%
20.93%
20.33%
53.61%

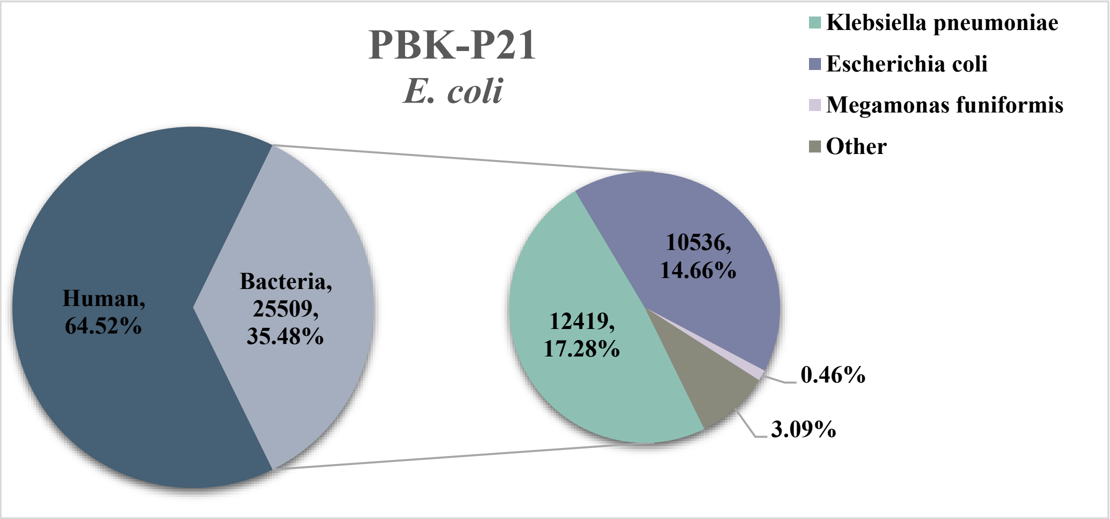
PBK-P21
E. coli
Klebsiella pneumoniae
Escherichia coli
Megamonas funiformis
Other
Human, 64.52%
Bacteria, 25509, 35.48%
10536, 14.66%
12419, 17.28%
0.46%
3.09%

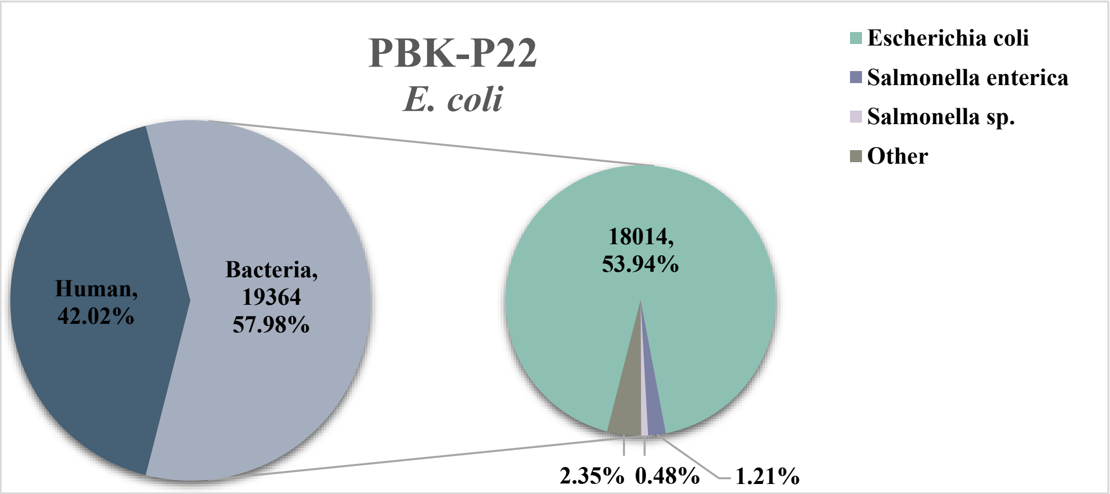
PBK-P22
E. coli
Escherichia coli
Salmonella enterica
Salmonella sp.
Other
Human, 42.02%
Bacteria, 19364 57.98%
18014, 53.94%
2.35%
0.48%
1.21%

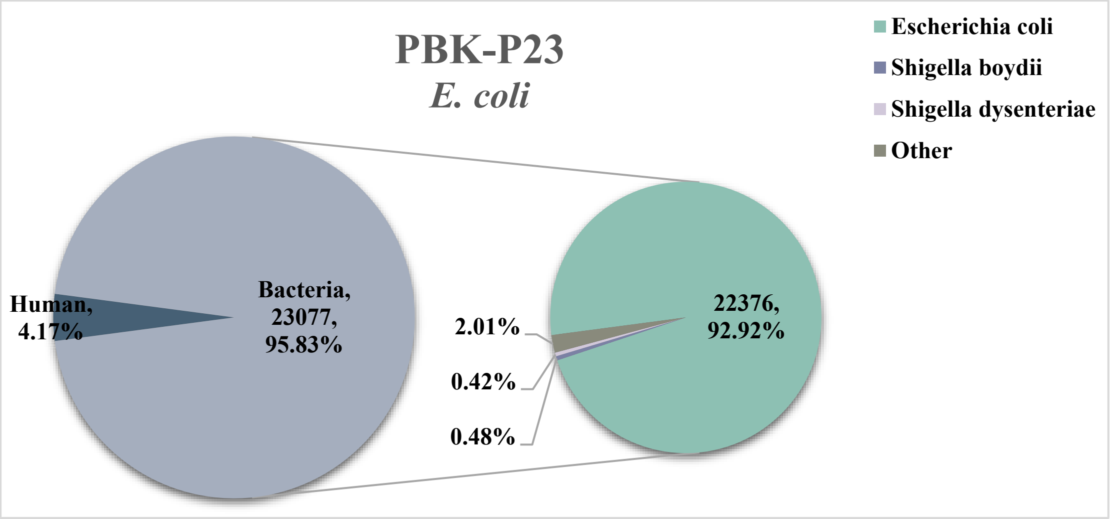
PBK-P23
E. coli
Escherichia coli
Shigella boydii
Shigella dysenteriae
Other
Human, 4.17%
Bacteria, 23077, 95.83%
22376, 92.92%
2.01%
0.42%
0.48%

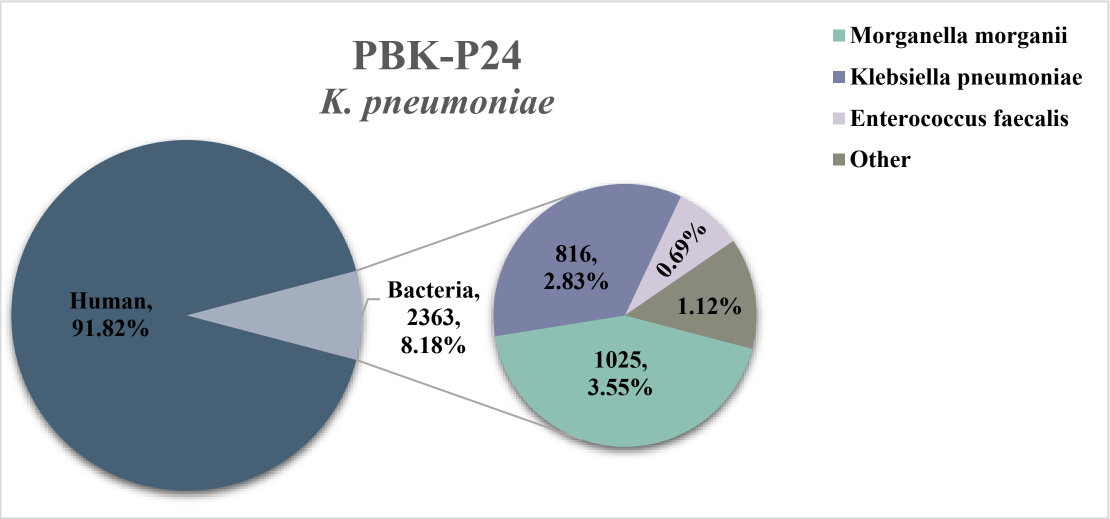
PBK-P24
K. pneumoniae
Morganella morganii
Klebsiella pneumoniae
Enterococcus faecalis
Other
Human, 91.82%
Bacteria, 2363, 8.18%
816, 2.83%
0.69%
1.12%
1025, 3.55%

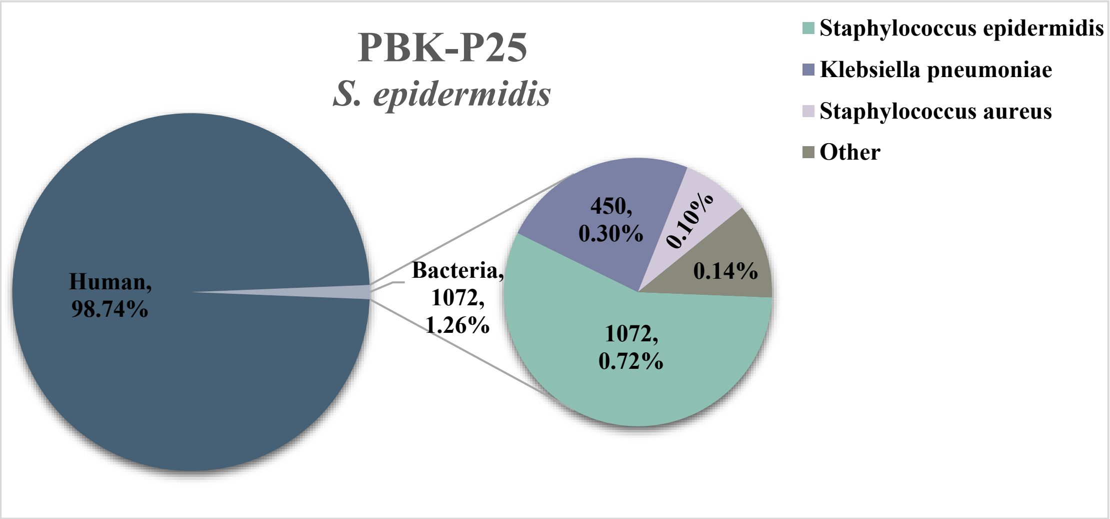
PBK-P25
S. epidermidis
Staphylococcus epidermidis
Klebsiella pneumoniae
Staphylococcus aureus
Other
Human, 98.74%
Bacteria, 1072, 1.26%
450, 0.30%
0.10%
0.14%
1072, 0.72%

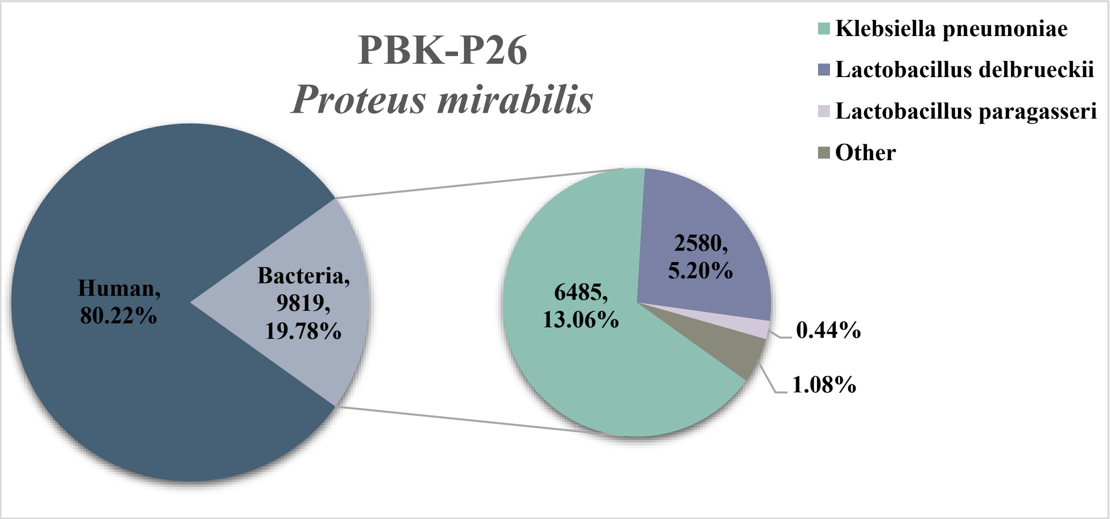
PBK-P26
Proteus mirabilis
Klebsiella pneumoniae
Lactobacillus delbrueckii
Lactobacillus paragasseri
Other
Human, 80.22%
Bacteria, 9819, 19.78%
2580, 5.20%
6485, 13.06%
0.44%
1.08%

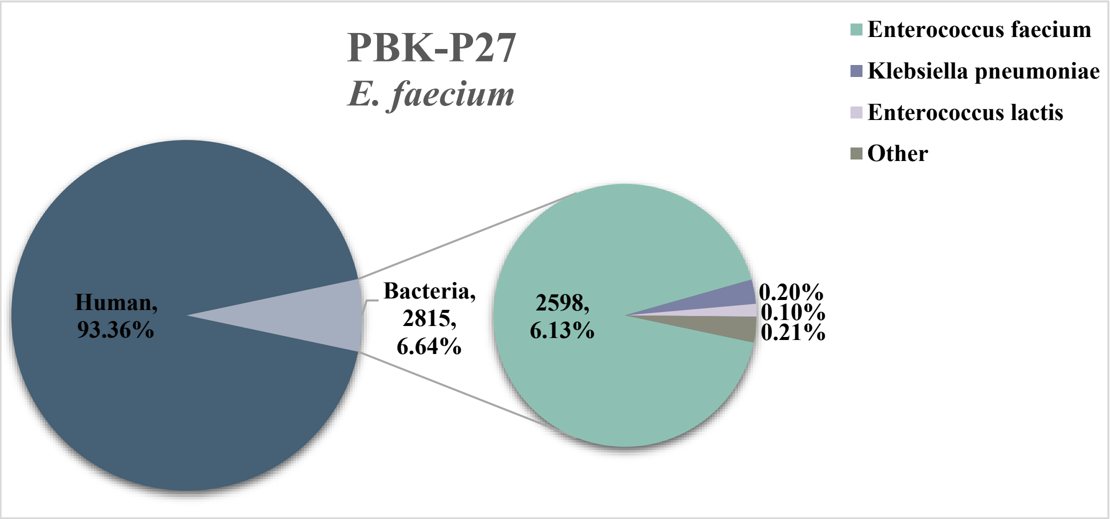
PBK-P27
E. faecium
Enterococcus faecium
Klebsiella pneumoniae
Enterococcus lactis
Other
Human, 93.36%
Bacteria, 2815, 6.64%
2598, 6.13%
0.20%
0.10%
0.21%

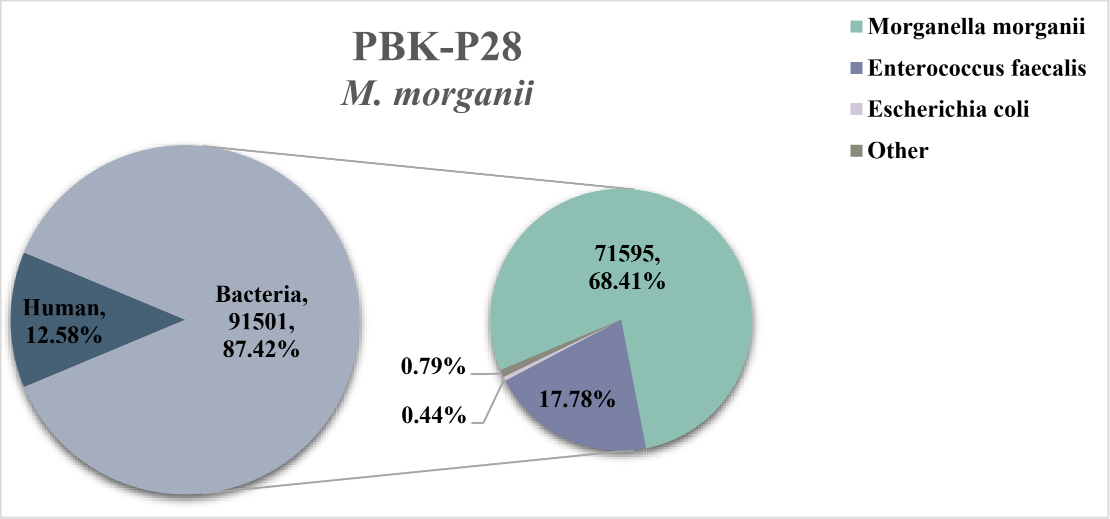
PBK-P28
M. morganii
Morganella morganii
Enterococcus faecalis
Escherichia coli
Other
Human, 12.58%
Bacteria, 91501, 87.42%
71595, 68.41%
0.79%
0.44%
17.78%

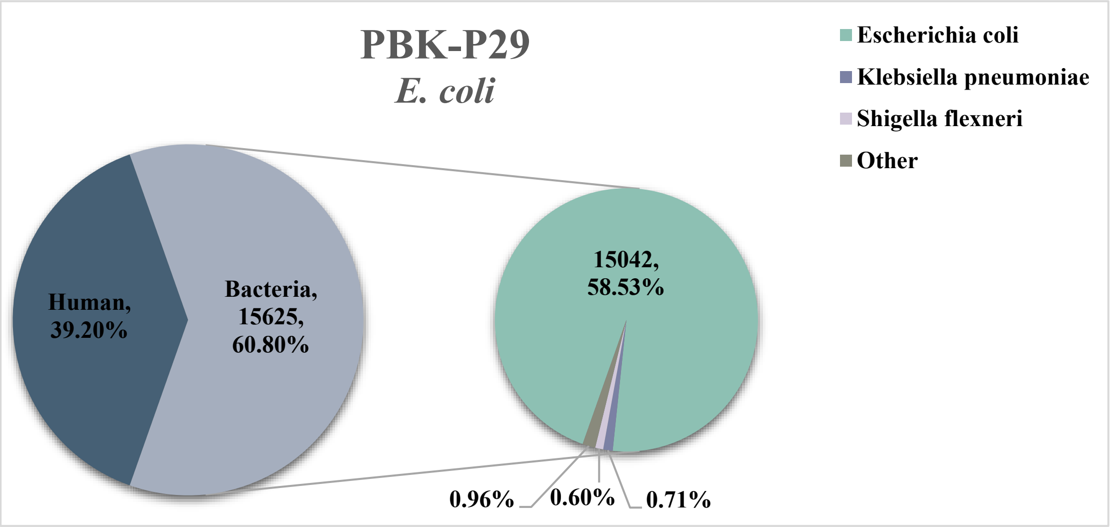
PBK-P29
E. coli
Escherichia coli
Klebsiella pneumoniae
Shigella flexneri
Other
Human, 39.20%
Bacteria, 15625, 60.80%
15042, 58.53%
0.96%
0.60%
0.71%

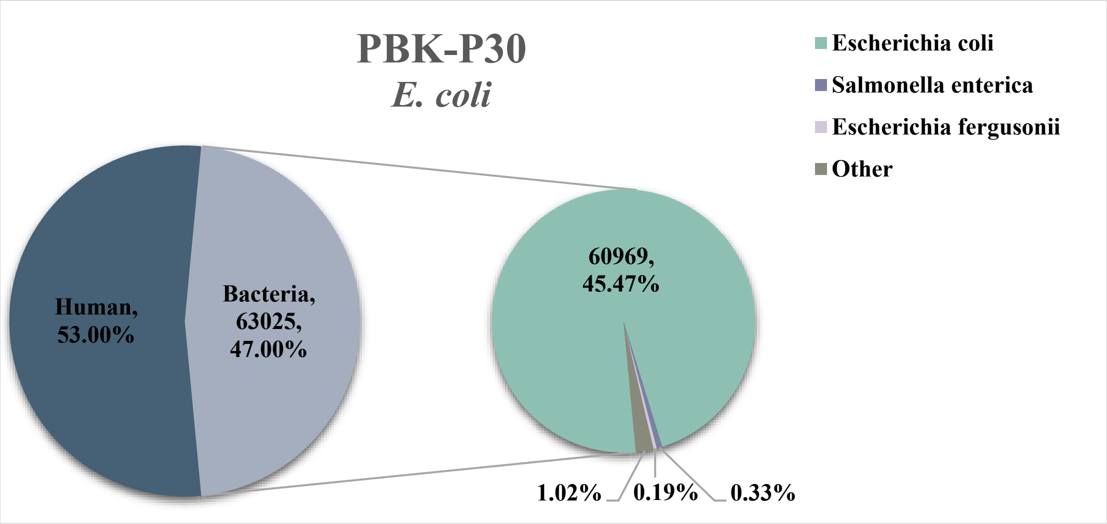
PBK-P30
E. coli
Escherichia coli
Salmonella enterica
Escherichia fergusonii
Other
Human, 53.00%
Bacteria, 63025, 47.00%
60969, 45.47%
1.02%
0.19%
0.33%

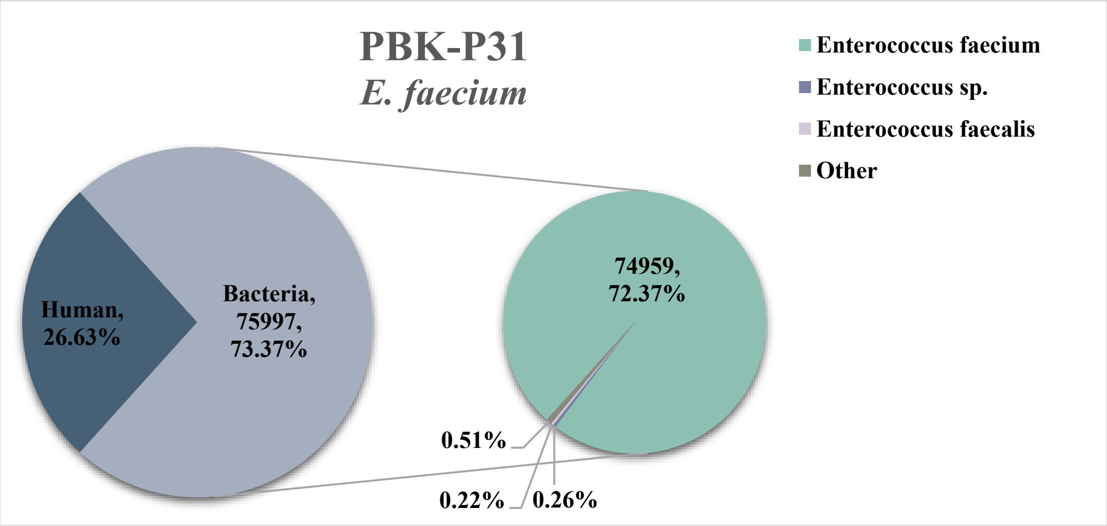
PBK-P31
E. faecium
Enterococcus faecium
Enterococcus sp.
Enterococcus faecalis
Other
Human, 26.63%
Bacteria, 75997, 73.37%
74959, 72.37%
0.51%
0.22%
0.26%

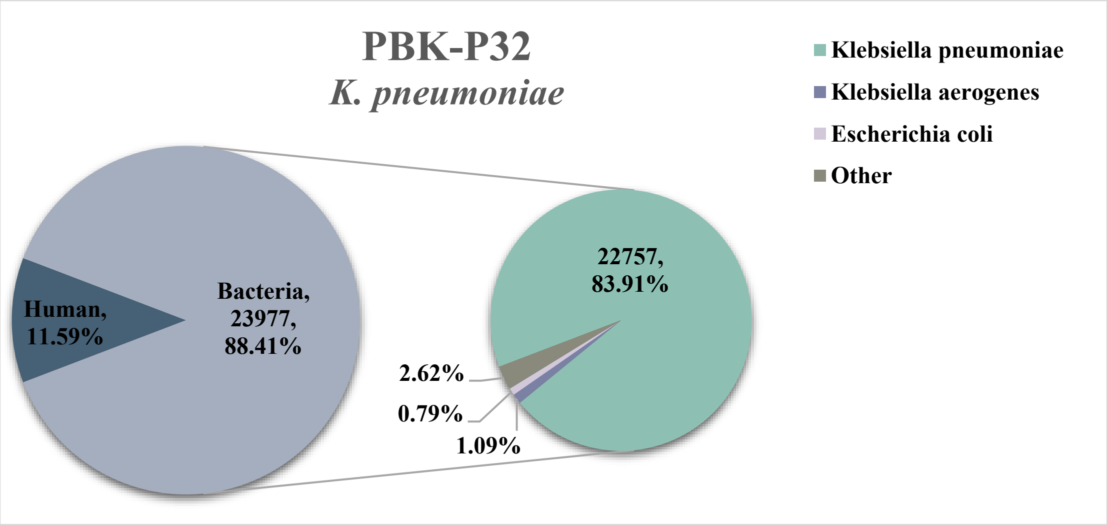
PBK-P32
K. pneumoniae
Klebsiella pneumoniae
Klebsiella aerogenes
Escherichia coli
Other
Human, 11.59%
Bacteria, 23977, 88.41%
22757, 83.91%
2.62%
0.79%
1.09%

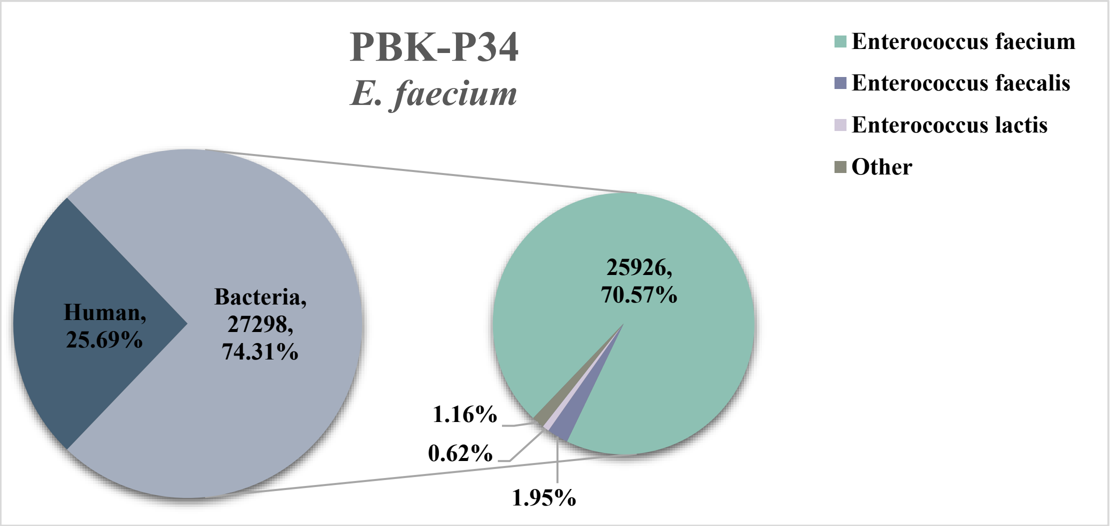
PBK-P34
E. faecium
Enterococcus faecium
Enterococcus faecalis
Enterococcus lactis
Other
Human,
25.69%
Bacteria,
27298,
74.31%
25926,
70.57%
1.16%
0.62%
1.95%

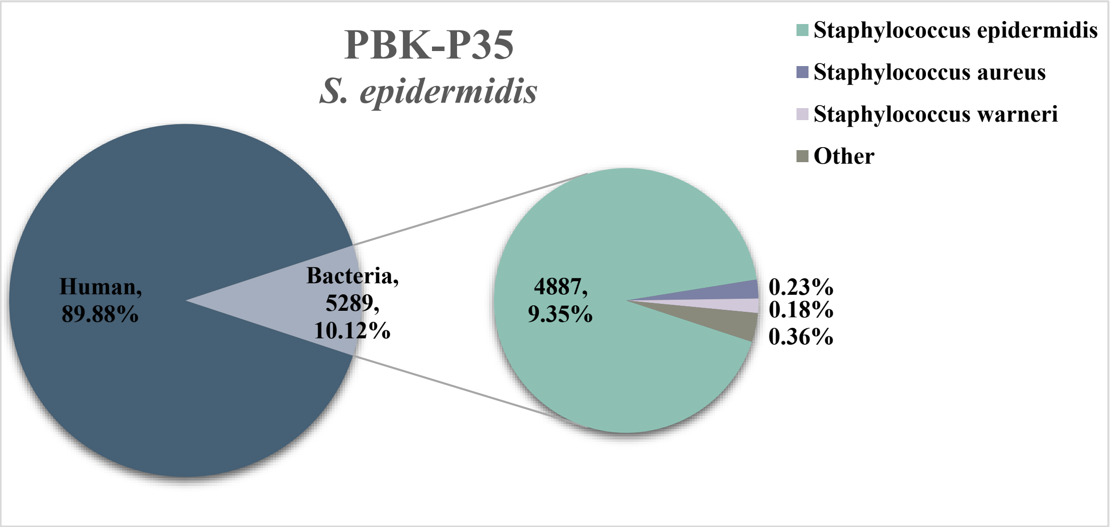
PBK-P35
S. epidermidis
Staphylococcus epidermidis
Staphylococcus aureus
Staphylococcus warneri
Other
Human,
89.88%
Bacteria,
5289,
10.12%
4887,
9.35%
0.23%
0.18%
0.36%

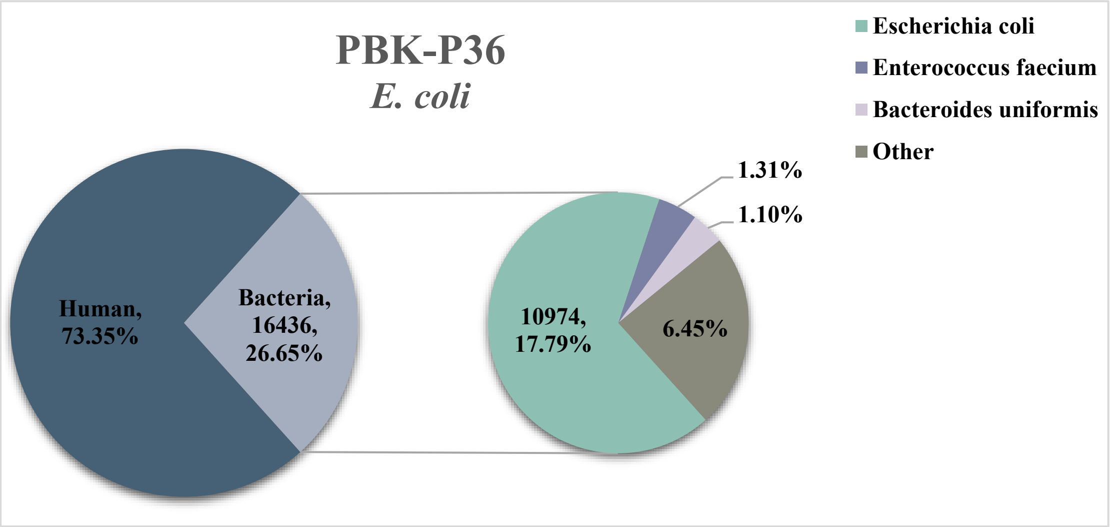
PBK-P36
E. coli
Escherichia coli
Enterococcus faecium
Bacteroides uniformis
Other
Human,
73.35%
Bacteria,
16436,
26.65%
10974,
17.79%
1.31%
1.10%
6.45%

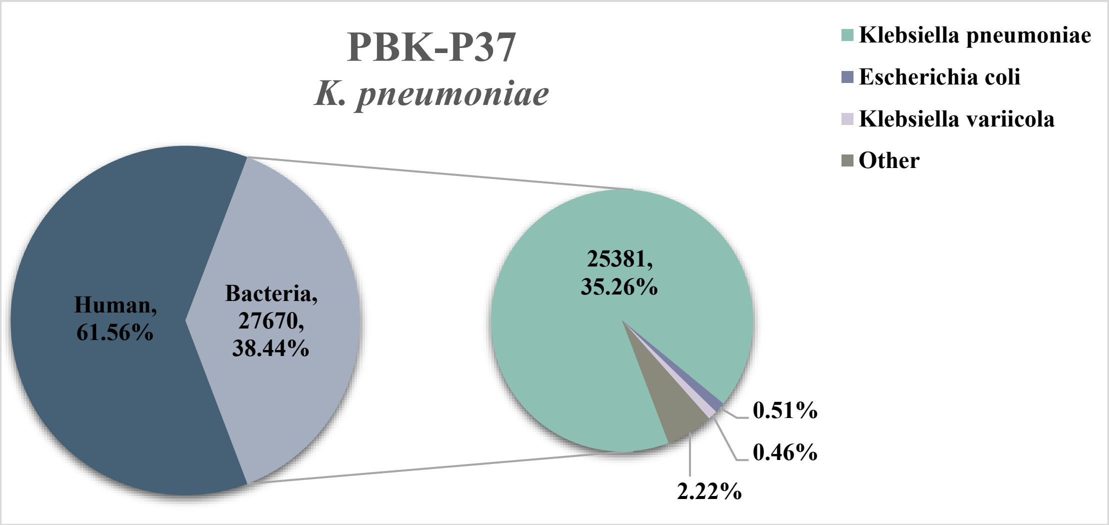
PBK-P37
K. pneumoniae
Klebsiella pneumoniae
Escherichia coli
Klebsiella variicola
Other
Human, 61.56%
Bacteria, 27670, 38.44%
25381, 35.26%
0.51%
0.46%
2.22%

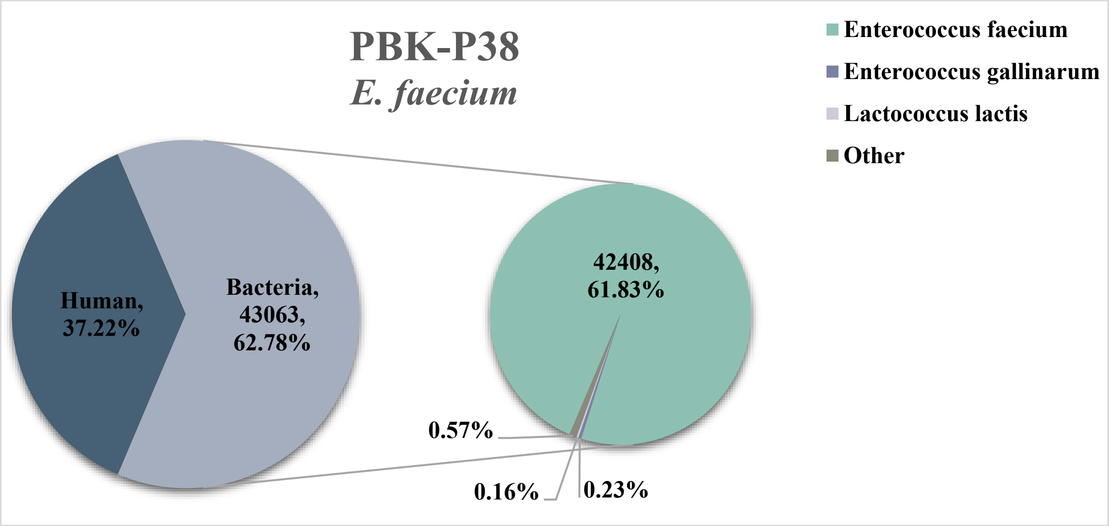
PBK-P38
E. faecium
Enterococcus faecium
Enterococcus gallinarum
Lactococcus lactis
Other
Human, 37.22%
Bacteria, 43063, 62.78%
42408, 61.83%
0.57%
0.16%
0.23%

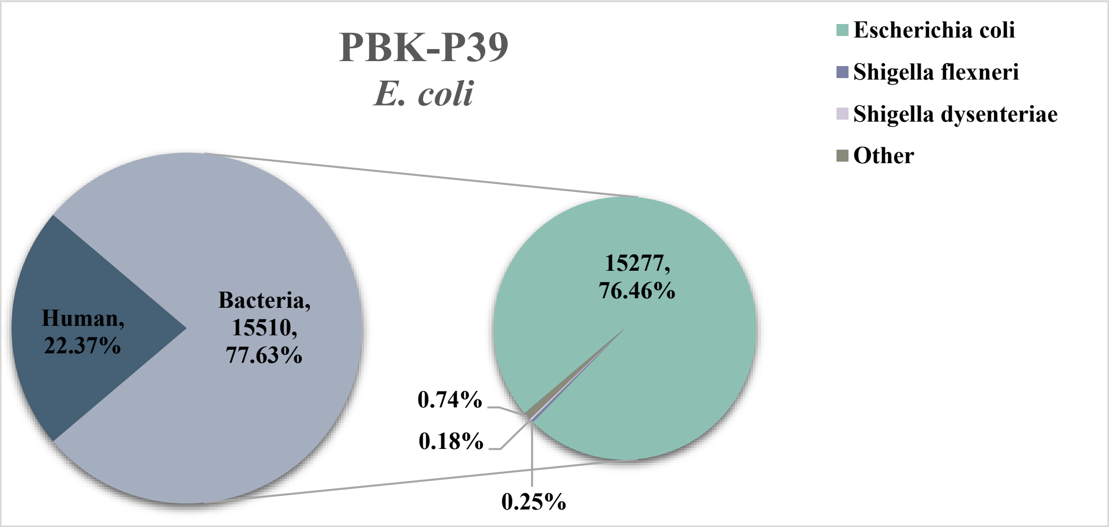
PBK-P39
E. coli
Escherichia coli
Shigella flexneri
Shigella dysenteriae
Other
Human, 22.37%
Bacteria, 15510, 77.63%
15277, 76.46%
0.74%
0.18%
0.25%

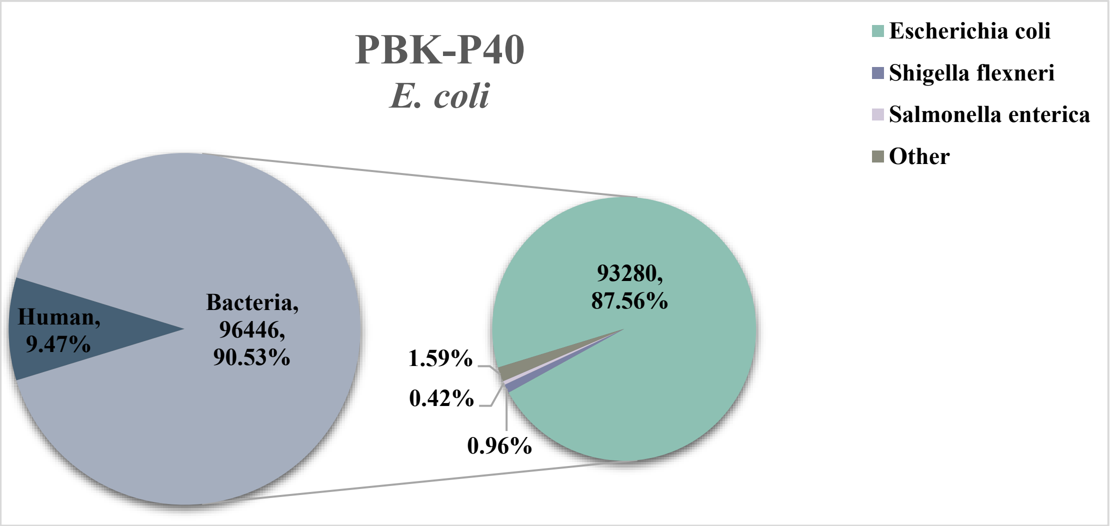
PBK-P40
E. coli
Escherichia coli
Shigella flexneri
Salmonella enterica
Other
Human,
9.47%
Bacteria,
96446,
90.53%
93280,
87.56%
1.59%
0.42%
0.96%

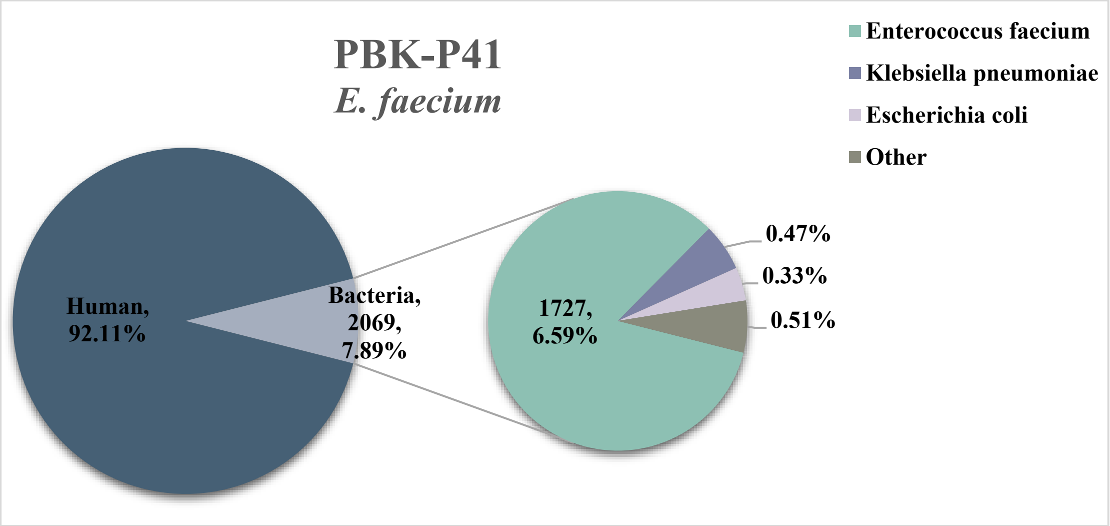
PBK-P41
E. faecium
Enterococcus faecium
Klebsiella pneumoniae
Escherichia coli
Other
Human,
92.11%
Bacteria,
2069,
7.89%
1727,
6.59%
0.47%
0.33%
0.51%

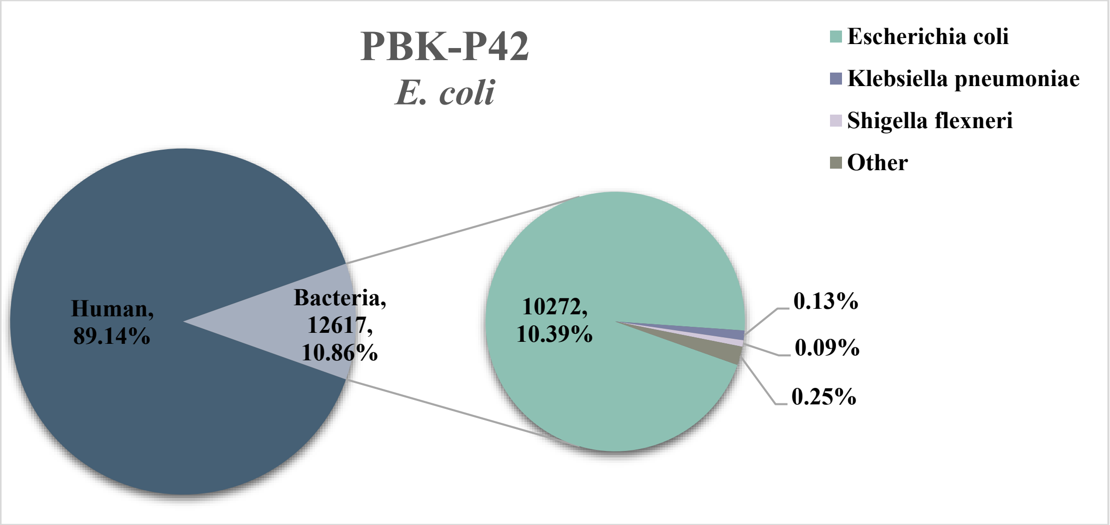
PBK-P42
E. coli
Escherichia coli
Klebsiella pneumoniae
Shigella flexneri
Other
Human,
89.14%
Bacteria,
12617,
10.86%
10272,
10.39%
0.13%
0.09%
0.25%

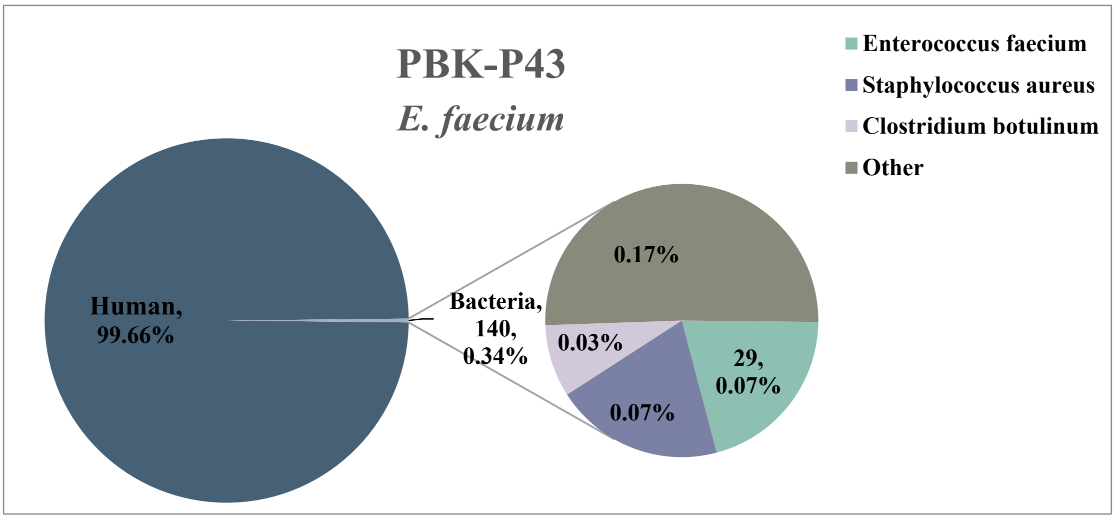
PBK-P43
E. faecium
Enterococcus faecium
Staphylococcus aureus
Clostridium botulinum
Other
Human, 99.66%
Bacteria, 140, 0.34%
0.17%
0.03%
29, 0.07%
0.07%

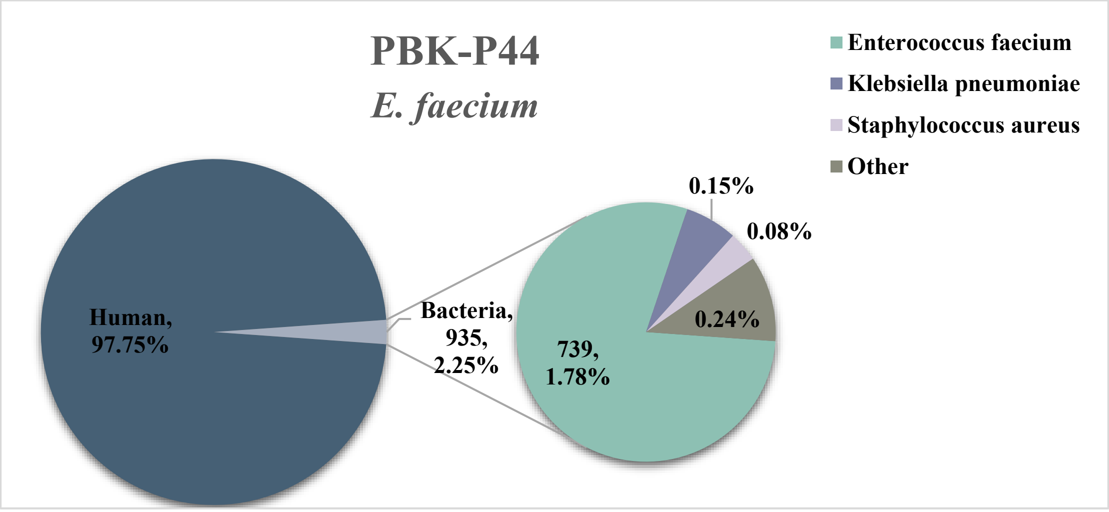
PBK-P44
E. faecium
Enterococcus faecium
Klebsiella pneumoniae
Staphylococcus aureus
Other
Human, 97.75%
Bacteria, 935, 2.25%
0.15%
0.08%
0.24%
739, 1.78%

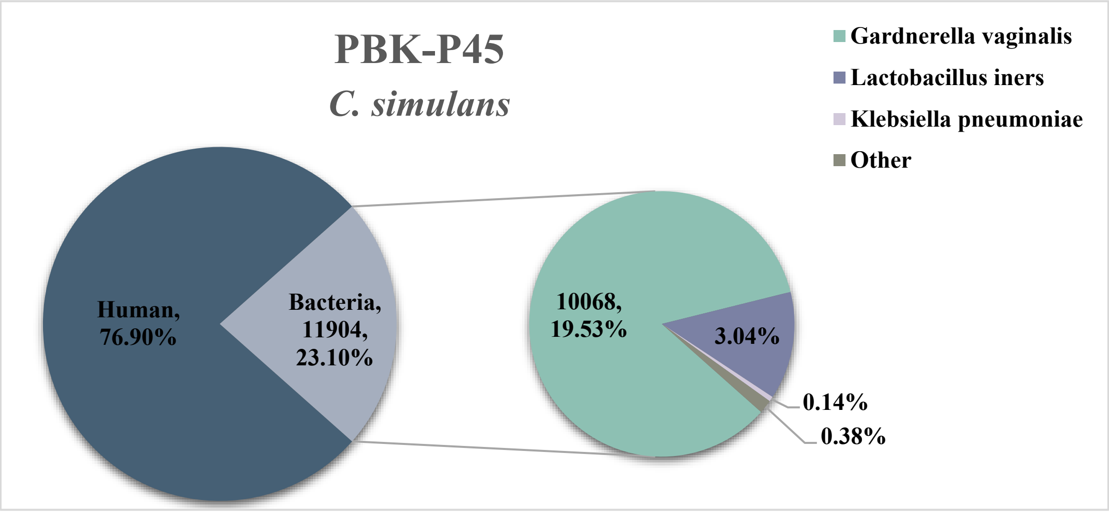
PBK-P45
C. simulans
Gardnerella vaginalis
Lactobacillus iners
Klebsiella pneumoniae
Other
Human, 76.90%
Bacteria, 11904, 23.10%
10068, 19.53%
3.04%
0.14%
0.38%

Supplementary Figure 1 | Pie charts demonstrating the taxonomic classification of reads for different library preparation methods and showing the top 3 bacteria with percentage distributions of sequencing reads. Different colors indicate different species, the title indicates the method of library preparation, sample number and culture-based result.
