# Supplementary figures and images for: Rapid Detection of Bacterial Pathogens and Antimicrobial Resistance Genes in Clinical Urine Samples With Urinary Tract Infection by Metagenomic Nanopore Sequencing
Source: Front Microbiol. 2022 May 17;13:858777. doi: 10.3389/fmicb.2022.858777 (PMC9152355; doi:10.3389/fmicb.2022.858777)

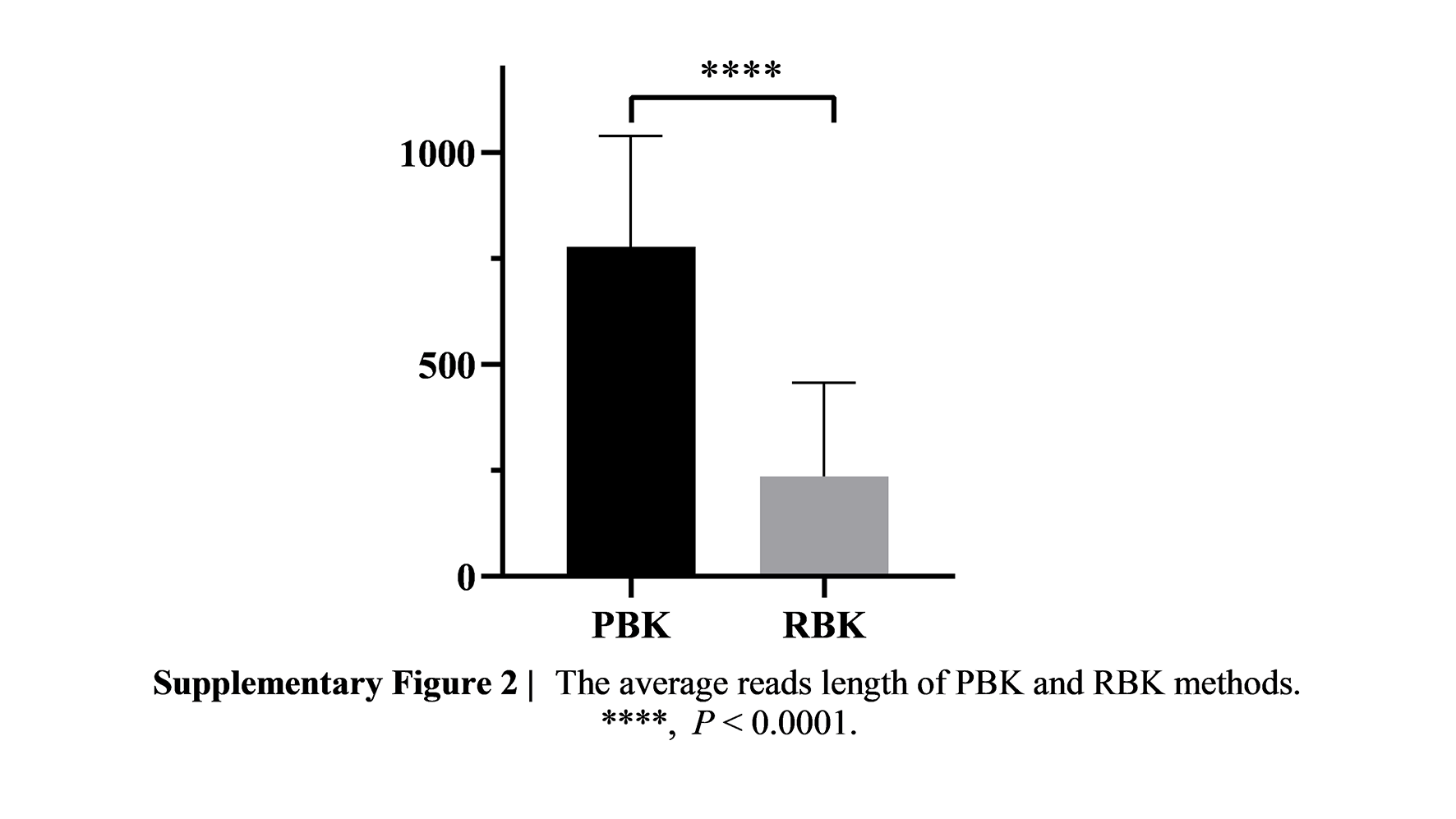

Supplement: Supplementary file 9 [file Image_2.tif]
